# Unevolved De Novo Proteins Have Innate Tendencies to Bind Transition Metals

**DOI:** 10.3390/life9010008

**Published:** 2019-01-09

**Authors:** Michael S. Wang, Kenric J. Hoegler, Michael H. Hecht

**Affiliations:** 1 Department of Chemistry, Princeton University, Princeton, NJ 08540, USA; msw5@princeton.edu; 2 Department of Molecular Biology, Princeton University, Princeton, NJ 08540, USA; kenhoegl@gmail.com

**Keywords:** protein design, novel metalloproteins, binary patterned amino acid sequences, prebiotic chemistry

## Abstract

Life as we know it would not exist without the ability of protein sequences to bind metal ions. Transition metals, in particular, play essential roles in a wide range of structural and catalytic functions. The ubiquitous occurrence of metalloproteins in all organisms leads one to ask whether metal binding is an evolved trait that occurred only rarely in ancestral sequences, or alternatively, whether it is an innate property of amino acid sequences, occurring frequently in unevolved sequence space. To address this question, we studied 52 proteins from a combinatorial library of novel sequences designed to fold into 4-helix bundles. Although these sequences were neither designed nor evolved to bind metals, the majority of them have innate tendencies to bind the transition metals copper, cobalt, and zinc with high nanomolar to low-micromolar affinity.

## 1. Introduction

Proteins that bind metals perform many of the essential functions necessary to sustain life [1]. Metalloproteins occur in every organism, and are required for a wide range of catalytic, structural, and signaling functions. Moreover, the amino acid sequences that bind metals and perform these functions are conserved across all three domains of life. These observations suggest that proteins capable of binding metals arose early in the history of life on earth [2,3,4].

Was metal binding by ancestral sequences a rare occurrence? Or do proteins have an innate tendency to bind metals? It is tempting to address these questions by studying natural proteins and assessing their abilities to bind various metals. However, extant natural proteins differ dramatically from the sequences that arose early in evolution. Current metalloproteins are the products of billions of years of selection for sequences that bind metals with high affinities and specificities. In contrast, the first ancestral sequences that bound metals may have done so with weak affinities, poor specificities, and/or overlapping binding sites.

In an attempt to gain insight into the innate metal binding of unevolved sequences, we focused on a collection of de novo proteins that were designed to fold, but were not explicitly designed to possess any binding or functional activities. The unevolved proteins used in this study were drawn from a combinatorial library of novel sequences, which were designed by binary patterning of polar and nonpolar residues to fold into 4-helix bundles [5,6,7]. These sequences differ fundamentally both from natural proteins and from de novo designed metalloproteins: In contrast to natural proteins, the binary patterned sequences are not biased by billions of years of selection for life-sustaining functions, and in contrast to rationally designed metalloproteins [8,9,10,11,12], the binary patterned sequences are not biased by explicit designs for metal binding sites.

To assess the abilities of these proteins to bind transition metals, 52 different sequences were incubated with transition metal ions immobilized on beads [13,14]. The metal binding ability of several of these proteins was further characterized by equilibrium dialysis and isothermal titration calorimetry. Although the sequences in this collection were neither designed nor evolved to bind metals, the majority of the de novo proteins exhibited some level of metal binding. Several of the novel sequences bound with affinities comparable to some natural metallo-peptides and proteins. The nanomolar affinity of some proteins for zinc is roughly comparable to human serum albumin (HSA), which has K_d_ ~ 3 × 10^−8^ [15]. These findings suggest that—at least in some cases—unevolved proteins have an innate tendency to bind transition metals.

## 2. Materials and Methods

**Gene Libraries, Strains, and Growth Conditions.** Genes for 3GL proteins were previously cloned into and expressed from a modified pCA24N vector containing a chloramphenicol (CAM) resistance cassette [16]. Protein expression is controlled by a T5 promoter and lac operator. 

Electrocompetent BW25113 cells were made according to standard procedures. Luria broth was supplemented with 30 μg/mL CAM. The working concentration of isopropyl β-d-1-thiogalactopyranoside (IPTG) was 100 µM. 

The 3GL plasmids were prepared in DNase free H_2_O to a final concentration of 50 ng/µL. Thus, 2 µL of the library were transformed into 100 μL of electrocompetent cells. Cells were then diluted 1:100 and 50 μL of cells were plated on LB/agar containing 30 μg/mL CAM. Plates were incubated at 37 °C for 12–16 h, after which single colonies became visible. 

**Screen for Soluble Expressers.** Overnight cultures were started from single colonies and grown for 12 h at 37 °C in LB containing 30 μg/mL CAM and 100 µM IPTG. Cell lysates were normalized to an OD_600_ of 1.0. Then, 1.5 mL of overnight culture was spun-down and re-suspended in 250 μL of BugBuster Protein Extract Reagent (Novagen, Madison, WI, USA). After 20 min incubation, samples were spun for 30 min at 13,000 rpm. 20 μL of the supernatants were diluted 1:1 with 2× Laemmli sample buffer and run on SDS-PAGE (Bio-Rad, Hercules, CA, USA). Proteins were visualized by staining with Coomassie blue.

Colonies that expressed high levels of protein were re-grown overnight in 5 mL LB containing 30 µg/mL CAM. The plasmid was purified from the overnight culture using a QIAprep Spin Mini kit (Qiagen). Sequencing of the 3GL gene was done by Sanger sequencing (Genewiz S., Plainsfield, NJ, USA). 

**Preparation of Immobilized Metal Beads.** Here, 80 μL of Sepharose beads chemically attached to iminodiacetic Acid (IDA) (Sigma-Aldrich, St. Lousi, MO, USA) were incubated with 100 μL of 250 mM CoCl_2_, CuCl_2_, or ZnCl_2_ for 20 min at room temperature. These were washed several times with wash buffer (50 mM Tris HCl, 500 mM NaCl, pH 7.6). Separation of beads and supernatants was done at low rpm (<3000) to ensure beads were not damaged.

**Assays for Metal Binding.** For this, 1.5 mL of overnight culture were spun-down and re-suspended in 250 μL of BugBuster Protein Extract Reagent (Novagen). After incubation for 20 min, samples were spun for 30 min at 13,000 rpm. Supernatants were incubated with iminodiacetic acid Sepharose Beads (Sigma-Aldrich) charged with Co^2+^, Cu^2+^, or Zn^2+^ for 1 h at 4 °C. The beads were washed 4 times for 4 min with stringent wash buffer containing 50 mM Tris HCl pH 7.6, 500 mM NaCl and the following concentrations of imidazole: 10 mM for cobalt, 50 mM for copper, and 15 mM for zinc. Beads were then suspended in 2× Laemmli sample buffer (Bio-Rad), heated to 95 °C for 5 min, and spun for 2 min at 13,000 rpm. Supernatants were normalized to the initially-loaded lysate volume and both were run on SDS-PAGE (Bio-Rad). Densitometry was analyzed using Image-Quant TL.

**Protein Expression and Purification.** BL21 *E. coli* cells expressing the protein of interest were grown overnight at 37 °C on LB plates containing 30 μg/mL CAM. Single colonies were picked and grown in liquid cultures (LB with 30 μg/mL CAM) for 12 h at 37 °C. Expression was induced at OD 600 ~0.5 by addition of 100 μM IPTG, and cells were allowed to grow for 16 h at 37 °C. Cells were lysed using an EmulsiFlex French press (Avestin, Ottawa, ON, Canada). Protein was purified in two steps. First, lysates were run over a His Trap HP nickel column (GE Healthcare, Chicago, IL, USA) washed with wash buffer (200 mM Na_2_HPO_4_, 500 mM NaCl, pH 7.4) and eluted with elution buffer (200 mM Na_2_HPO_4_, 500 mM NaCl, 250 mM imidazole, pH 7.4). Even without a His-tag, the proteins bind the nickel column, presumably because of the high number of histidine residues on their surfaces. HisZero was the only protein that did not stick to the nickel column, and was instead first purified by Anion Exchange on a HiTrap Q HP column (GE Healthcare) with start buffer (200 mM Na_2_HPO_4_, 100 mM NaCl, pH 7.6) and elution buffer (200 mM Na_2_HPO_4_, 1.5 M NaCl, pH 7.6) in a linear gradient. Second, fractions containing the desired protein were run over a HiLoad 26/60 superdex 75 size exclusion column (GE Healthcare) in buffer (10 mM Tris, 100 mM NaCl, pH 7.6). Protein purity and identity were verified by HPLC (Agilent 100 system, Agilent Zorbax 300SB-C18 column) followed by ESI-MS (Agilent 6210 TOF LC/MS).

**Equilibrium Dialysis.** A Pierce™ Rapid Equilibrium Dialysis (RED) Device, 8 kDa MWCO (ThermoFisher, Waltham, MA, USA) was used to analyze metal binding. Here, 5 μM of pure protein in standard buffer (10 mM Tris, 100 mM NaCl, pH 7.6) were placed in chamber A of the apparatus, and increasing amounts of CoCl_2_, CuCl_2_, or ZnCl_2_ diluted in standard buffer were placed in chamber B. The dialysis apparatus was shaken at 800 rpm for 2 h at room temperature to reach equilibrium. An aliquot of the metal solution was then removed from chamber B and an equal volume of higher-concentration metal solution was added. From a series of such measurements, the curve of bound vs. free metal was determined. Measurements were not taken above concentrations where the protein precipitated, as determined by spinning down separate solutions of proteins incubated with metals. All equilibrium dialysis experiments were performed in triplicate.

The concentration of metal in chamber B was measured using 500 µM 4-(2-pyridylazo)resorcinol (PAR) (Thermo Fischer Scientific) in standard buffer. This was done by external calibration using dilutions of TraceCERT^®^ metal standards (SigmaAldrich) in standard buffer. The linear relationship between binding and absorbance (495 nm) enabled determination of the concentration of metal in chamber B. The limit of detection (LoD = 3σ) was 60 nM for Co^2+^, 120 nM for Cu^2+^, and 75 nM for Zn^2+^. Bound metal was identified as the difference between total metal present in the dialysis and the free metal measured. All data were baselined against a buffer blank run without protein. Calculations were performed using standard protocols, and binding curves were fit using least-squares fitting.

**Isothermal Titration Calorimetry (ITC).** A MicroCal PEAQ-ITC calorimeter (Malvern Panalytical, Malvern, UK) was used to verify several of the binding curves. Each titration was performed at 25.0 °C, with the reaction cell containing 250 µL of protein (10 or 20 µM) in standard buffer (10 mM Tris, 100 mM NaCl, pH 7.6), and the injection syringe filled with Co(II), Cu(II), or Zn(II) metal solutions (100, 250, or 500 µM depending on stoichiometry) in standard buffer. The enthalpy evolved upon metal binding was monitored by proxy of the energy needed to hold the temperature constant. Each titration experiment was performed using 18 injections of 2 μL with 8-s duration and a 120 to 160-s interval between injections. Reference power of 41.9 µW, high-feedback mode, and a stirring speed of 1000 rpm were used for all experiments. All data were analyzed by using the MicroCal PEAQ-ITC analysis software. To obtain the binding enthalpies, the observed enthalpy values were corrected for the enthalpy of dilution observed when the protein was saturated with metal.

## 3. Results

### 3.1. A Collection of Unevolved De Novo Proteins

**Figure 1:**
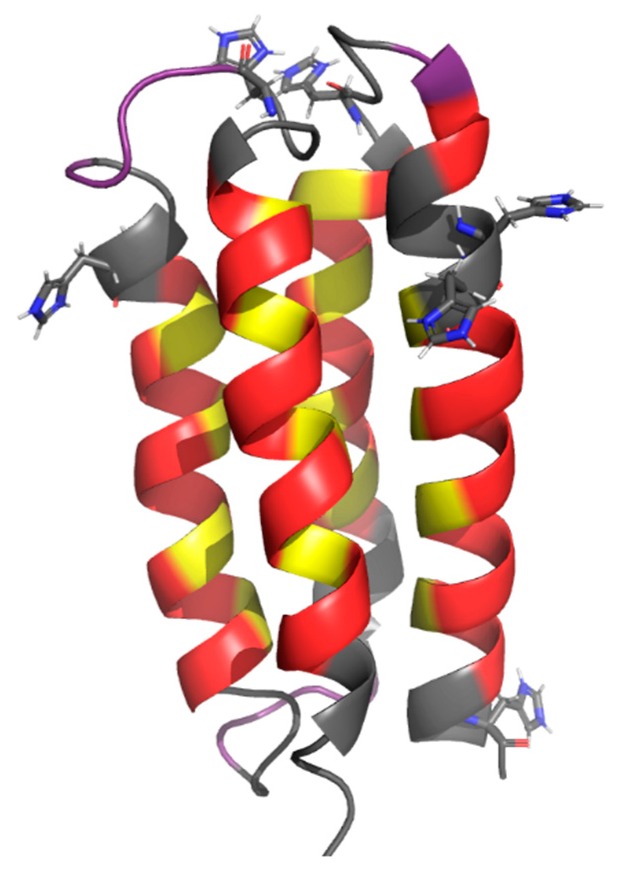
NMR structure of the 4-helix bundle protein, S-824 (PDB code 1P68) [18]. Polar residues are colored red, nonpolar residues are yellow, and nonhelical residues are purple. Residues that were conserved in the design of this library are in grey, with conserved histidines shown explicitly.

We previously described a strategy that uses binary patterning of polar and nonpolar residues to design libraries of novel α-helical proteins [5,6,7]. In this approach, the sequence locations of polar and nonpolar residues are specified explicitly; however, the exact identities of the residues at each position are not specified, and are varied combinatorially. Combinatorial diversity is enabled by using degenerate DNA codons to construct a collection of synthetic genes encoding the library of novel proteins. The codon NTN (N = A,T,C,G) is used to encode five nonpolar amino acids (Phe, Leu, Ile, Met, Val), and the alternate codon VAN (V = A,C,G) is used to encode six polar amino acids (His, Glu, Gln, Asp, Asn, Lys). We have created several libraries of binary patterned proteins designed to fold into 4-helix bundles. Proteins from these libraries were characterized biophysically, and several 4-helix bundle structures were determined at high resolution [17,18,19,20]. An example protein structure, S-824, is shown in Figure 1.

From these collections, we isolated a novel protein (called ConK) that rescues *E. coli* from lethal concentrations of copper [21]. This last finding suggests that novel proteins, which have not undergone evolutionary selection, can nonetheless have significant impacts on metal binding and/or homeostasis 

For the current study, we wished to assess the ability of proteins—chosen arbitrarily from this library of unevolved sequences—to bind transition metals. To facilitate rapid screening, we chose a subset of proteins from our third generation library (3GL) [7] that expressed high levels of soluble protein.

To prepare this collection, we transformed the 3GL plasmid library into *E. coli* BW25113 and plated on LB agar supplemented with chloramphenicol, as described previously [16]. Single colonies were chosen arbitrarily and grown overnight in the presence of 100 µM IPTG to induce expression. Cell numbers were normalized, and clarified lysates were run on SDS-PAGE and stained with Coomassie Brilliant Blue. If a dark band was seen at approximately 12 kDa (the mass of a 3GL protein of 102 residues), the clone was included in the sub-library. Each member was provided the name naïve metal binder (NMB), followed by a number. Thus, NMB 5 is the fifth well-expressed soluble protein isolated. Overall, approximately two-thirds of the clones expressed high levels of soluble protein. In this manner, 52 proteins were selected for further characterization.

### 3.2. Construction of Weak Metal Binders as Negative Controls

Preliminary screens suggested the majority of our de novo proteins bind transition metals. Therefore, in order to establish a baseline for proteins that do not bind transition metals, we designed two new sequences based on sequence S-824, a protein from a binary patterned library, which was shown previously to form the stable well-ordered 4-helix bundle structure shown in Figure 1 [16,17]. 

The first putative non-binder was designed by mutating all six histidines in the combinatorial regions of S-824 to other polar residues. Histidine is a known metal-binding residue, and we wished to use a sequence devoid of histidines in the combinatorial regions as one negative control. This variant was named S-824-HC (HisConserved). This sequence still contains the six conserved histidines in the turn and helical regions that were held constant in the library design.

The second negative control was made using the Rosetta Sequence Tolerance protocol [22] to replace the six remaining histidines in the S-824-HC variant with residues that do not bind metals. This variant was named S-824-HZ (HisZero). These new proteins allowed us to establish two lower limits for metal binding from de novo proteins: the HisConserved shows the lower limit available to the library that was tested, while the HisZero shows the binding available if histidines are removed entirely from the design. Sequences of all proteins used in this study are shown in Supplementary Material Table S1.

### 3.3. Screening Novel Proteins for Binding to Transition Metals

**Figure 2:**
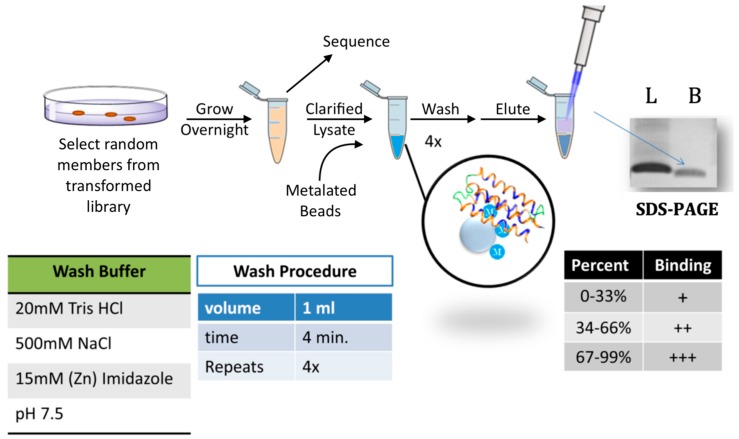
Screen for metal binding. Cells expressing a de novo protein were grown overnight in the presence of isopropyl β-d-1-thiogalactopyranoside (IPTG). Plasmids were then sequenced and cells were lysed. Clarified lysates were incubated with iminodiacetic acid (IDA) Sepharose beads that had been charged with a metal cation. The bound proteins underwent several stringent washes (see “wash buffer” and “wash procedure” panels). Samples were then eluted by boiling in SDS sample buffer, run on SDS-PAGE, stained with Coomassie, and quantified by densitometry. The amount of protein loaded (L) was compared to the amount that remained bound (B) to the beads. Based on the percentage bound, proteins were binned into three groups (bottom right panel).

Immobilized metal affinity chromatography (IMAC) resins were used to screen the sub-library of 52 well-expressed soluble de novo proteins for binding to transition metals (Figure 2). When using IMAC resins, several metal and chelator combinations are possible. We chose to test three transition metals known to play roles in a range of biological systems: cobalt, copper, and zinc. We immobilized these metals on the resin using the tridentate chelator, iminodiacetic acid (IDA). We chose this chelator because it leaves the most metal ion coordination sites available, but still binds transition metals strongly enough to ensure the metal would remain bound to the bead during stringent washes. IDA Sepharose beads (Sigma Aldrich) were charged with Co^2+^, Cu^2+^, or Zn^2+^.

Single clones from the sub-library were grown overnight in the presence of 30 μg/mL IPTG to induce expression. Clarified lysates from these cultures were incubated with the metalated beads, and then washed with a buffer containing salt and imidazole. A high concentration of salt (500 mM NaCl) was used to shield non-specific electrostatic interactions with the resin. Imidazole concentrations for the washes were established by control experiments with the HisConserved protein described above. HisConserved was removed from the beads by washes with imidazole concentrations of 10 mM for cobalt, 50 mM for copper, and 15 mM zinc. In contrast to HisConserved, the HisZero protein did not stick to the metalated beads. 

**Figure 3:**
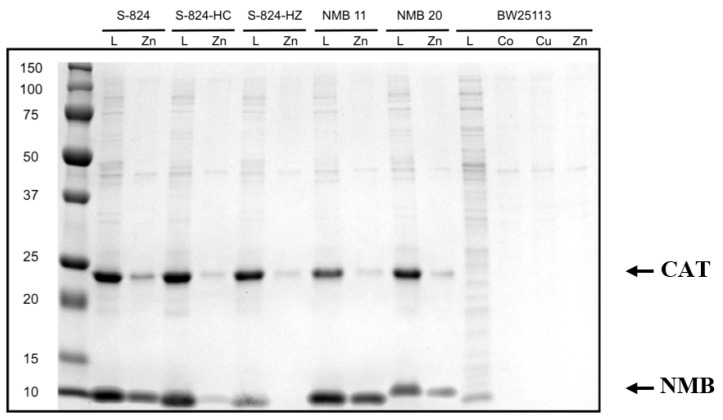
SDS-PAGE comparing bound protein to loaded protein. A representative gel showing protein binding to metalated beads. Each gel was run with protein S-824 as a positive binding control and with S-824-HC as a negative binding control. A sample of the clarified lysate that had been loaded onto the bead (L) was run next to the sample of protein that remained bound to the bead following several stringent washes (metal). Our novel metal binding proteins (labeled “NMB”) run near 12 kDa. Densitometry designated NMB 11 as a moderate zinc binder (++) while NMB 20 was designated a weak zinc binder (+). The four rightmost lanes correspond to the *E. coli* strain without the expression plasmid. The band running near 25 kDa is chloramphenicol acetyl transferase (CAT), which is also encoded on the expression plasmid.

**Figure 4:**
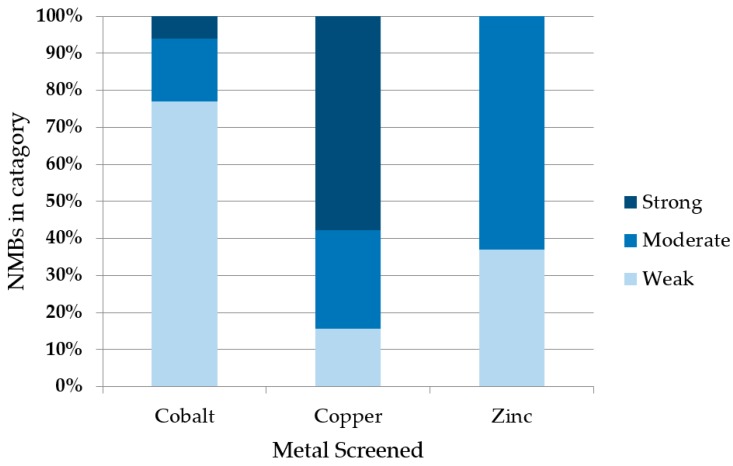
Summary of binding by 52 de novo proteins to three transition metals. Co^2+^, Cu^2+^, or Zn^2+^ ions were immobilized on IMAC beads and incubated with cell lysates expressing 52 different de novo proteins. Binders were binned into three groups based on the fraction of protein that remained bound to the metalated bead after stringent washes with imidazole. The majority of NMBs bound copper strongly, zinc moderately, and cobalt weakly.

To assess metal binding, we compared the amount of de novo protein that remained bound to the metalated bead (after stringent washing) to the amount initially loaded. Samples were run on SDS-PAGE and bands were quantified by densitometry. Examples are shown in Figure 3. Based on the fraction of protein that remained bound to the metalated resin, the 52 sequences were grouped into three classes: If <33% of the protein remained bound to the bead, the protein was designated a non-binder or a weak binder (+); if 33–66% of the protein remained, it was classified as a moderate metal binder (++); and if 66–100% remained bound, it was classified as a strong binder (+++). The majority of proteins bound cobalt weakly, zinc moderately, and copper strongly (Figure 4). Despite the stringency of imidazole washes being varied to match the Irving-Williams series (10 mM for cobalt, 15 mM for zinc, and 50 mM for copper), the identified ordering still corresponds with the affinities expected from this series [23]. A full list of proteins and their screened relative binding affinities for the three metals is shown in Supplementary Material Table S2.

To compare our findings with these de novo proteins to natural *E. coli* proteins, we repeated the procedure using *E. coli* cells not containing an expression vector. As shown in Figure 3, under these conditions, the vast majority of endogenous *E. coli* proteins do not bind divalent metals on the resin.

### 3.4. Estimation of Binding Affinities

Binding to metalated beads showed that many of our unevolved proteins have some affinity for the tested transition metals. However, binding to metalated beads provides little information about affinity constants, and no information about binding stoichiometry.

To estimate the metal binding affinity and stoichiometry of the novel proteins, we used equilibrium dialysis. In this technique, a selectively permeable membrane divides two chambers. The purified protein is added to chamber A, and increasing concentrations of a metal salt (e.g., ZnCl_2_) are added chamber B. The membrane allows metal ions (but not protein) to flow between chambers until equilibrium is established. By measuring the final concentration of metal in chamber B, one can determine how much metal bound to the protein in chamber A.

Because our proteins were neither evolved nor designed to bind metals, we anticipated that binding might not be limited to single well-defined sites; and multiple sites with similar affinities might occur on a single protein. Therefore, we analyzed our data to estimate the stoichiometry of binding, and to calculate an “apparent” dissociation constant (K_d,app_), which represents the ensemble binding affinity for multiple sites on a protein, rather than an exact K_d_ for each individual site. The procedure for calculating stoichiometry and K_d,app_ from the equilibrium dialysis measurements is summarized in Supplementary Material Equation (1)–(8).

**Figure 5:**
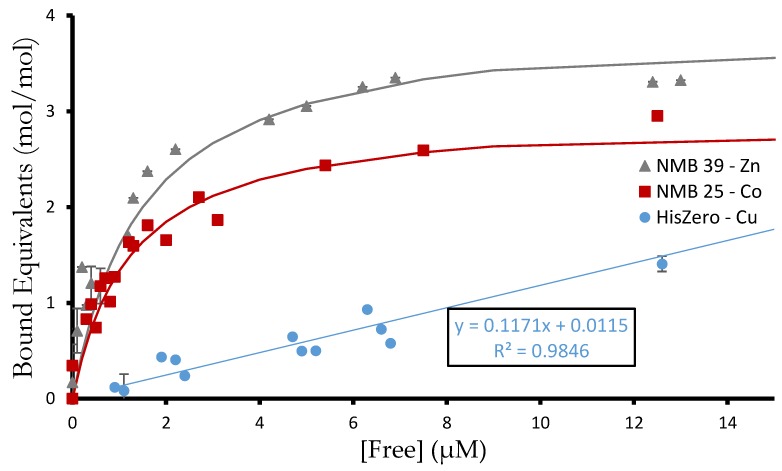
Binding of de novo proteins to different metals. Equilibrium dialysis was used to determine the binding coefficient, [Metal bound]/[Protein total]. As protein is titrated with Co^2+^, Cu^2+^, or Zn^2+^, the K_d,app_ is the concentration of metal ion at which half of the metal binding sites on the protein are occupied. Shown are three representative binding curves from the 27 experiments. NMB 39 binds 4 equivalents of zinc with K_d,app_ ≅ 1 µM. NMB 25 binds three equivalents of cobalt with K_d,app_ ≅ 1.25 µM. The binding of HisZero to copper shows linear behavior proportional to free metal, which is characteristic of weak nonspecific electrostatic interactions.

We measured metal binding for six NMB proteins and three control proteins for cobalt, copper, and zinc (Figure 5). These NMB proteins were chosen because they represented a range of apparent metal affinities in the initial screen. For example, NMB 11 seemed to prefer zinc over other metals, whereas NMB 20 had the opposite trend and disfavored zinc binding. The three controls, S-824, S-824-HisConserved, and S-824-HisZero, were also assayed. Binding data for all proteins are shown in Table 1, with binding curves in Supplementary Material Figure S1.

The equilibrium dialysis experiments identified affinities ranging from 200 nM (NMB 37 binding copper) to 2.3 µM (NMB 24 binding cobalt). They also ranged in stoichiometry from one binding event (NMB 39 binding copper) to four (NMB 39 binding zinc). Thus, the stoichiometry can differ for a given protein binding different metals. This suggests certain sites bind metals selectively.

Several of the de novo proteins bind non-integer equivalents of metal. This suggest that in some cases, a metal ion may be shared between two protein monomers. We also note that partial occupancy of the metal coordination sphere by the de novo protein does not preclude simultaneous binding by solvent and/or buffer molecules. In particular, Tris is known to complex Cu(II) with millimolar affinity [24]. Buffer interactions in direct competition with protein and/or in the formation of ternary complexes with metal and protein may cause the calculated K_d,app_ to underestimate the true metal binding affinity.

The two negative controls reveal further nuances of binding. The HisConserved variant bound at least one equivalent of each metal ion, showing that the conserved histidine residues are sufficient for some of the binding observed in the NMB proteins. In contrast, the HisZero variant did not bind Co^2+^ or Cu^2+^ with significant affinity, and bound one equivalent of Zn^2+^ with affinity comparable to other NMB proteins. As HisZero shows the binding possible from combinatorial regions without histidine, it is likely that carboxylic acid residues in the binary pattern are sufficient to bind Zn^2+^. Overall, both negative controls exhibited poor metal binding and established the minimum binding for library proteins.

To confirm the affinities and stoichiometries measured by equilibrium dialysis, we also measured these properties for NMB 39 S-824 and S-824-HisZero by Isothermal Titration Calorimetry (ITC). The ITC results (Supplementary Materials Table S3 and Figure S2) corroborate the equilibrium dialysis measurements in order of magnitude, but not always stoichiometry. For example, both techniques showed that S824 binds three equivalents of Zn(II), and HisZero binds Zn(II), but not Co(II) or Cu(II). However, equilibrium dialysis differed from ITC by identifying 4 binding events for NMB 39 binding to Zn(II), while ITC identified only 1.5 binding events.

The difference in apparent stoichiometry between equilibrium dialysis and ITC probably reflects the different properties measured by these two techniques: Equilibrium dialysis relies on a colorimetric indicator to assay metal concentrations. In contrast, ITC records the ∆H of binding. For NMB 39, the measured ∆H of binding was on average −44.5 kJ/mol. Smaller enthalpies associated with additional binding sites might be dwarfed by this signal. Consequently, these sites may not be seen, thereby diminishing the apparent stoichiometry observed by ITC.

A surprising counterpoint is that for S-824 binding to Co(II) and NMB39 binding to Cu(II), ITC detects *more* binding events than does equilibrium dialysis. The commonality between these cases is that they are the least enthalpically-favored metal binding of those tested (i.e., lowest ∆H relative to other metals). While ITC may detect these binding sites, they may reach equilibrium more slowly in the dialysis experiments, and so be undetected. Despite occasional differences, these two orthogonal techniques both detect multiple binding sites with low micromolar affinities.

In general, separate binding events are observed only when these events have distinctly different affinities. Consequently, two distinct binding events were detected by ITC only in the case of S-824 binding Co. For this reason, S-824 binding Co had a higher affinity measured by ITC than suggested by the IMAC screen or by equilibrium dialysis. Because these separate events can be difficult to observe, we chose to report the binding values as the “apparent” average of multiple events.

Though it is difficult to correlate the solution experiments with the bead experiments, both suggest ready metal binding. A possible cause of discrepancies between the techniques is that the beads rely on binding to a tridentate IDA, which occupies half the coordination sites of the metal, while the equilibrium dialysis experiments present hydrated metals with all sites potentially available. Another difference is that the stringent washes in the IMAC screen are calibrated to wash away the HisConserved protein and may remove weakly bound proteins.

## 4. Discussion

Metal binding proteins are ubiquitous in nature. Nearly half of all proteins contain a metal [25], and a survey of 1371 different enzymes with known 3-D structures estimated that 47% required a metal for function and 41% contained a metal at their catalytic centers [26]. Because they play essential roles in biology and are conserved across all forms of life [1], it has been suggested that metals—and cofactors containing metals—played key roles in the first replicating entities that gave rise to life on earth. These considerations suggest that the earliest ancestral amino acid sequences may have been selected, in part, for their intrinsic abilities to bind metal ions. 

As a proxy for ancient proto-proteins, we studied a library of synthetic de novo proteins with no evolutionary history; and assessed their abilities to bind transition metals. Because design of a metal-binding site was not a part of the library design, and so must arise by chance, we considered the possibility that binding would be rare among our unevolved sequences. Consequently, when planning this project, we expected to require high-throughput methods, such as phage display [27], to find rare needles in a large haystack. However, as described above, our experimental result showed that artificial metalloproteins are not rare, and occur frequently in our collection of de novo proteins.

The observed frequency of metal binding suggests that—at least in some cases—unevolved proteins possess an innate ability to bind transition metals. This supports the suggestion that the early selection of the 20 proteinogenic amino acid side chains may have been influenced by a biological requirement for polymers that bind transition metals [28].

The de novo proteins in our collection have no evolutionary history. Yet they are likely to differ from ancestral natural proteins in several ways: First, the library proteins are all α-helical, while natural proteins contain α-helices, β-strands, and non-repetitive structures. Second, because the combinatorial library was constructed using the degenerate codons VAN and NTN to encode polar and nonpolar residues, respectively, cysteine, which is encoded by TGC and TGT, does not occur in our proteins. Yet, cysteine is an important metal-binding residue in nature. Third, the de novo proteins are rich in exposed His- Glu-, and Asp- residues. Thus, although unbiased by evolutionary history, our binary pattern proteins are biased to contain an abundance of the metal binding residues His, Asp, and Glu. In particular, the de novo proteins in our collection have an abundance of solvent-exposed histidines; an average of 12 histidines per 102-residue sequence. The HisConserved control protein contains six histidines, while some proteins in the collection contain as many as 18. The abundance of histidine residues results from the binary code strategy, which specifies histidine as one of only six residues that can occupy surface exposed positions on all four α-helices [5,6,7].

While the abundance of histidines in our proteins makes them different from most proteins in the current biosphere (which contain ~2.3% histidine), ancestral sequences may have also been rich in histidine. It has been proposed that histidine was one of the earliest genetically encoded amino acids, and was originally encoded by all four CAN codons. According to this proposal, glutamine arose later, and adopted the CAA and CAG codons by “codon capture” [29]. If this was indeed the case, then ancestral proteins may have contained abundant histidines, and like our unevolved de novo proteins, may have bound transition metals more readily than their current descendants.

Glutamine may have evolved as an alternative to histidine, in part, to limit the tendency of primordial sequences to bind non-specifically (and promiscuously) to metals and metal-containing cofactors. This is consistent with our finding that current *E. coli* proteins—which have a lower percentage of histidines than our de novo proteins—also have a lower tendency to bind metalated resins (Figure 3).

Much as codon capture by glutamine may have decreased the abundance of histidines in the *sequences* of modern proteins, the scarcity of solvent-exposed histidines in the *structures* of modern proteins may result from negative selection against the type of non-specific metal binding that occurs so readily among the binary patterned de novo structures.

The IMAC resin screens for sites that allow a metal to simultaneously bind both the resin and the protein. In modern proteins, however, most histidines occur in buried or partially buried active sites [30,31]. They are not abundant on protein surfaces, and rarely occur as exposed clusters that would lead to promiscuous metal binding. Consequently, our IMAC screen may isolate natural metalloproteins only if they (1) are in their *apo* form, (2) have binding sites that are not deeply buried, and (3) do not bind so tightly as to abstract the metal from the resin. Likewise for the de novo proteins, the IMAC screen would fail to identify NMBs with buried sites or with a tightly bound metal that co-purifies with the protein following cell lysis.

Removing solvent-exposed histidines in the negative controls, HisConserved and HisZero, decreased binding to the metalated resin (Figure 2). Therefore, we considered the possibility that binding to metalated resin might be a simple function of the number of histidines in each of the de novo sequences. To test this possibility, we compared the number histidines (and the metal binding carboxylates, Glu and Asp) per 102-residue sequence against the fraction of each protein retained by the metalated resin. No correlation was observed (Supplementary Material Figures S3–S5). Nonetheless, the failure of the histidine-free control HisZero to bind the metalated resin suggests, broadly, that the binding observed in the library relies on histidine residues more than Asp or Glu. 

**Figure 6:**
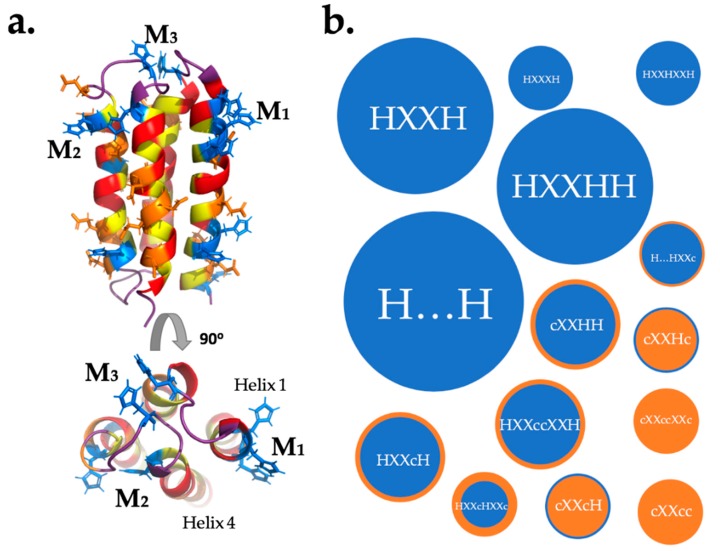
Possible binding sites in a binary-patterned 4-helix bundle. (**a**) NMR structure of S824 (PDB code 1P68). Polar residues are in red, nonpolar residues are in yellow, potential metal-coordinating residues are colored blue (His) or orange (Asp, Glu). The figure shows three ways metal ions (M) could be coordinated: by residues in one helix (M_1_) between two helices (M_2_) or by loop residues (M_3_). (**b**) Frequency of possible binding motifs in S-824 and all NMB proteins, where H is His and c is either carboxylic acid Glu or Asp. Mutants of S-824 were not included as their binding sites are a subset of those in S-824. The size of a bubble is determined by the abundance of that motif, while color is dictated by abundance of H (blue) against c (orange). Ellipsis (…) indicate a large separation of residues on different helices.

The lack of correlation between the number of histidines per sequence and binding to the metalated beads indicates that binding of transition metals by unevolved de novo proteins—like binding by natural proteins—depends on the relative positioning (not just the frequency) of metal binding side chains in the three-dimensional structure of the protein. Assuming that many of these sites are the same sites that bind the free metal, we modelled the relative locations of His, Asp, and Glu side chains on the predicted structures of our NMB proteins. Assuming that a binding site requires two or more metal binding residues to be close in space, we used QUARK ab initio structure prediction [32] to visualize which residues might be positioned in ways that could lead to a primitive metal binding site (Figure 6a). The manual search considered both ligand strength (H > D,E) and the distance between residues. A list of possible binding sites is shown in Table 2. In those cases where a putative site has several nearby metal-binding residues, it is not possible to predict which will actually be involved in binding. For example, in the sequence H_1_XXH_4_H_5_ the metal could be coordinated between residues 1 and 4, 1 and 5, or all three. The possible binding motifs for protein S-824 and the NMB proteins assayed by equilibrium dialysis are plotted by frequency of occurrence in Figure 6b.

Mutagenesis of S-824 to remove histidines revealed two important sites. The first is a conserved binding site between residues H_24_ and H_74_, shown as M_3_ in the Figure 6. Based on the HisConserved construct, this should bind 1 equivalent of each metal. This conserved sequence is responsible for the large abundance of H…H motifs in Figure 6. The second is that the observed binding of zinc to HisZero could arise from a DXXED motif. This uses three carboxylic acid residues, which are hard bases, and would be expected to bind Zn^2+^, which is a harder metal than Co^2+^ or Cu^2+^. Both these binding insights should hold true for all the NMB proteins: The H_24_…H_74_ motif is conserved and motifs with only carboxylic acid residues, and no histidines, might selectively bind zinc but not the other metals. Further studies involving mutagenesis of each NMB will be required to confirm which of the predicted sites are responsible for the observed binding.

It is also noteworthy that several of the conserved histidines participate in approximately half the proposed binding. Thus, H_83_ and H_84_ are responsible for the common motif HXXHH, shown in Figure 6. Likewise, H_101_ is a participant in three of the six HXXH motifs. Because these conserved histidines appear in all of the sequences in our library, these findings can also be viewed as analogous to the evolutionary improvement of ancestral metal binding sequences.

As noted in the preceding section, many of the putative binding sites include conserved residues. Yet, 12 of the 27 proposed binding sites do *not* involve any conserved residues. They result from a semi-random combinatorial exploration of sequence space, and thereby highlight the high frequency at which metal binding can occur among sequences that were neither designed nor evolved to bind metals.

The simple model for binding shown in Figure 6 does not lead to occupancy of the entire coordination sphere of the metal. Therefore, the proposed binding sites are incomplete and binding sites that bridge two protein molecules are possible. This is consistent with our experimental results, which indicate that some NMB proteins may form ternary complexes, and in some cases, precipitate proteins that are brought together. Other studies have suggested an important role for metals in protein-protein interfaces [33]. Our work supports such a role for metals in the early evolution of protein-metal complexes. This also emphasizes the evolutionary counterpoint: ternary protein-metal complexes can promote amyloid formation [34].

The apparent dissociation constants (K_d,app_) for the six characterized NMB proteins reveal high-nanomolar to low-micromolar affinities. This range is similar to natural proteins like human serum albumin (HSA), but dramatically different from the tight binding measured for many proteins; superoxide dismutase, for example, has sub-picomolar affinity for zinc [15]. This affinity is, however, comparable to some de novo proteins explicitly designed to bind metals [11].

The tight metal binding affinities of many modern proteins is necessary in the metal-limited environment of the modern cell, which requires that evolved natural proteins must have high affinities and specificities to be metalated correctly in vivo. In contrast, our unevolved de novo proteins, with their moderate affinities and promiscuous specificities, would not be metalated in the highly evolved cytoplasm of *E. coli* [25].

Most of the proteins in our collection bound copper stronger than zinc, which they bound stronger than cobalt (Figure 4). This suggests, not surprisingly, that innate unevolved binding correlates with the Irving-Williams series. While some metal preference is indicated by both affinity and stoichiometry, the apparent binding is both promiscuous and ubiquitous; quite different from modern natural proteins. Because binding different metals can allow natural proteins to exhibit new functions [35], the permissive binding observed in these unevolved proteins might serve as a source from which selectivity and function can arise following natural or artificial selection.

In summary, the findings reported here suggest that some level of metal binding is not difficult to achieve. Instead, binding occurs frequently in collections of sequences containing abundant His, Asp, and Glu residues, even though the positions and relative geometries of these residues were not specified a priori. These results show that the ability of proteins to bind transition metals requires neither eons of natural selection or years of rational/computational design. Instead, it appears that unevolved de novo proteins have innate tendencies to bind transition metals.

## Figures and Tables

**Table 1:** **Equilibrium dialysis results for metal binding for six NMB proteins and three controls.** The apparent binding constants (K_d,app_), and the stoichiometry of metal ions per protein (max bound equivalents) are shown. “-” indicates very weak electrostatic binding, as discussed in the text.

Protein	Metal	K_d,app_ (nM)	Max Bound Equivalents	Protein	Metal	K_d,app_ (nM)	Max Bound Equivalents
S-824	Co^2+^	700	1.5	NMB 24	Co^2+^	2300	3
	Cu^2+^	700	1.5		Cu^2+^	1000	3
	Zn^2+^	1000	3		Zn^2+^	600	2
S-824-	Co^2+^	900	1.5	NMB 25	Co^2+^	1200	3
HC	Cu^2+^	300	1		Cu^2+^	1200	2
	Zn^2+^	600	2		Zn^2+^	500	4
S-824-	Co^2+^	–	–	NMB 37	Co^2+^	1600	3
HZ	Cu^2+^	–	–		Cu^2+^	200	2
	Zn^2+^	1500	1		Zn^2+^	1200	4
NMB 11	Co^2+^	600	3	NMB 39	Co^2+^	700	2
	Cu^2+^	800	3		Cu^2+^	600	1
	Zn^2+^	300	4		Zn^2+^	1000	4
NMB 20	Co^2+^	300	1.5				
	Cu^2+^	600	2				
	Zn^2+^	800	3				

**Table 2:** Possible binding sites on the assayed proteins. Conserved residues are in blue text. Binding sites involving a conserved residue are placed in a “C” row while independent binding sites are in an “I” row. Stoichiometry is from equilibrium dialysis.

Protein		Binding Motifs				Stoichiometry		
						Co^2+^	Cu^2+^	Zn^2+^
S-824	C	H_24_…H_74_	H_20_…H_31_XXD_33_	H_80_XXH_83_H_84_	H_94_XXE_97_E_98_XXH_101_	1.5	1.5	3
	I	D_7_XXE_10_D_11_XXE	E_42_XXH_45_D_46_					
S-824-HC	C	H_24_…H_74_	E_97_E_98_XXH_101_			1.5	1	2
	I	D_7_XXE_10_D_11_XXE						
S-824-HZ	I	D_7_XXE_10_D_11_XXE				0	0	1
NMB 11	C	H_24_…H_74_	H_31_D_32_XXH_35_	H_83_H_84_XXH_87_	H_98_XXH_101_	3	3	4
	I	H_59_XXH_62_	E_66_XXD_69_E_70_					
NMB 20	C	H_24_…H_74_	H_97_H_98_XXH_101_			1.5	2	3
	I	H_14_XXH_17_H_18_	E_42_XXD_45_H_46_					
NMB 24	C	H_24_…H_74_	H_31_XXE_34_E_35_	H_83_H_84_XXH_87_	H_97_H_98_XXH_101_	3	3	2
	I	H_39_XXD_42_E_43_XXH_46_						
NMB 25	C	H_24_…H_74_	H_80_XXH_83_H_84_			3	2	4
	I	H_11_… H_43_	H_31_D_32_XXH_35_	H_43_XXH_46_	H_59_XXH_62_			
NMB 37	C	H_24_…H_74_	H_83_H_84_XXD_87_	H_98_XXH_101_		3	2	4
	I	D_7_XXD_10_H_11_	H_31_XXE_34_H_35_XXE_38_					
NMB 39	C	H_24_…H_74_	H_31_D_32_XXE_35_	D_80_XXH_83_H_84_	H_95_XXH_98_XXH_101_	2	1	4
	I							

## Supplementary Materials

The following are available online at http://www.mdpi.com/2075-1729/9/1/8/s1, Figure S1: Binding Curves.
