# Supplementary material for: Unevolved De Novo Proteins Have Innate Tendencies to Bind Transition Metals
Source: Life (Basel). 2019 Jan 9;9(1):8. doi: 10.3390/life9010008 (PMC6463171; doi:10.3390/life9010008)
Supplement: Supplementary file 1 [file life-09-00008-s001.pdf]

Supplementary Information: Unevolved *De Novo* Proteins Have Innate Tendencies to Bind Transition Metals

Protein Sequence Information:

Table S1. Amino acid Sequences of 52 well expressed Naïve Metal Binders (NMB) proteins. The controls S-842, S-824-HC, and S-824-HZ are also included. All proteins are 102 amino acids long. The four designed α-helices have combinatorial sequences of polar and nonpolar residues, while regions at the ends of the helices are constant. Sequences are presented in the following format: For S824, M is the first residue, G is the 51st, G is the 52nd and R is the 102nd. Conserved regions are underlined, deliberate mutations are colored red. The combinatorial assembly of the library is imperfect so some of the tested proteins are slightly different than designed – these errors are indicated in blue.

PROTEIN	AMINO ACID SEQUENCE
S-824	MYGKLNDLLEDLQEVLKNLHKNWHGGKDNLHDVDNHLQNVIEDIHDFMQGG<br>GSGGKLQEMMKEFQQVLDELNNHLQGGKHTVHHIEQNIKEIFHHLEELVHR
S-824 - HC	MYGKLNDLLEDLQEVLKNLQKNWHGGKDNLHDVDNKLQNVIEDIQDFMQGG<br>GSGGKLQEMMKEFQQVLDELNNHLQGGKNTVHHIEQNIKEIFKQLEELVHR
S-824 - HZ	MYGKLNDLLEDLQEVLKNLQKNWGGGKDNLKDVDNKLQNVIEDIQDFMQGG<br>GSGGKLQEMMKEFQQVLDELNNFLQGGKNTVKQIEQNIKEIFKQLEELVKR
NMB 1	MYGKLNKMIKNFQNMLQEFNKNWHQGEDNLHDLKKKMEQLVNDLHNFMQGG<br>EDRGKLQDVLKNMHEIMDQINNHLQSSQDTVHHFKEDLQELFKNLNHLVHR
NMB 2	MYGKLNQLMQELDKLVKHFHKNWHRHDNNLLDLDHNLENMLHDLEDFMQGR<br>HRNGKLQNVLNHINDLLNQLDNHLQRSRKTVHHLNHHMQDLLQNLHNLVHR
NMB 3	MYVKLNDILNKLEHLLHELNKNWHRHGRNLHDLDHHLENFIDQFQHFMQGK<br>RRKGKLQELLEHVNQILNQLHNHLQENDKTVHHIEDKLDEMLDHMEHLVHR
NMB 4	MYGKLNQFFEELHKIVDNLNKNWHRQNSNLHDIDKQLEEFLKNLKHFMQGN<br>SRDGKLQQLLEQLKQILDDIDNHLQDNDQTVHHLDQELQHMLNELNDLVHR
NMB 5	MYGKLNKMLKHMQELLKELDKNWHGERGNLHDIKHEMEQLFQHLKHFMQGH<br>SEKGKLQELMNKLNQIINKLDNHLQGDRGTVHHFNKHLNQFMDELNNLVHR
NMB 6	MYGKLNNIMDNMQHLIEKLHKNWHGDRRNLHDMKHQLENFMEQLQHFMQGG<br>QNGGKLQDLLDQMHKFIQQLDNHLQREGDTVHHLHKQIHNVMDNFHHLVHR
NMB 7	MYGKLNQFMKDMQQMIEEIHKNWHGRHRNLHDMKQQIHQLFNELEHFMQGG<br>EGEGKLQELIKQIQQMLNKFDNHLQDERETVHHFEQDFHKIIHQLHHLVHR
NMB 8	MYGKLNKVLNQLDDVLNKFHKNWHRGRGNLHDMDHDFQEMLXQFDDFMQGN<br>NQEGKLQHLLEQLHELIEQMHNHLQNNSQTVHHIHQKLNHFIHXLNHLVHR
NMB 9	MYGKLNKMIHQMEEFLQELDKNWHGRKKNLHDLEDQLKHLLQHMEDFMQGK<br>DRKGKLQDILNQFQHVLQHLDNHLQDDGHTVHHIKEHIDQLLNDLNNLVHR
NMB 10	MYGKLNKVLKNLEKLLEHIDKNWHRGGGNLHDLDHHMEKVLKQVIHEIENF<br>MQGRGKLQEIMNELQNILKNMDNHLQEKNNTVHHVEEEVEDLIEQLHHLVHR
NMB 11	MYGKLNEMVNQMKDMVDKINKNWHQGSKNLHDINHQIDQIIQDVEHFMQGH<br>NENGKLQHLVHKMNELLDELHNHLQEDNHTVHHFEHQLQKLVDQLQHLVHR
NMB 12	MYGKLNEIMKQFKHLLDKLHKNWHGSQQNLHDLQEHIKDLMDKMDNFMQGN<br>GNKGKLQHVLKNMHEIMDQINNHLQSSQDTVHHLDNKLKEILQHLHHLVHR
NMB 13	MYGKLNDMFQKIHQLMEELDKNWHEWHQNLHDLQQNFQQLFDQFDHFMQGS<br>NNHGKLQDILDHLKQFMNEFNNHLQEGGGTVHHVEQHFDEFFKKMKHLVHR
NMB 14	MYGKLNQLFHNLNEIVEDLNKNWHRESRHLHEFEDELHQLVKHFHHFMQGH<br>KNEGKLQDIVKQLDKLFRDLDNHLQRKDDTVHHLHHQLNKLLEQLDNLVHR
NMB 15	MYGKLNKMLHKIQQLLHDIDKNWHHEERNLHDLNQDLKDMLHNLQHFMQGR<br>EEGGKLQDLLKELDQMLEQLNNHLQSRNSTVHHLDQNLNKVMEQLQDLVHR
NMB 16	MYGKLNDMLHHMEDFMQELNKNWHNSGSNLHDLEEKVDNLMHNVDNFMQGN<br>DNHGKLQHVVQKLQDVLNELDNHLQGNGHTVHHLHNQLEQIMKDLHNLVHR
NMB 17	MYGKLNKLLQQMHKLFNNMNKNWHHRHRNLHDLQEQMQQILENFQDFMQGQ<br>KENGKLQHLLNKIKHFLEQLDNHLQRGEETVHHLQQEFKQLLNLHDNLVHR
NMB 18	MYGKLNELFEQIEDILKHMHKNWHKGQRNLHDLQEHLKQVINQLNHFMQGH<br>GGHGKLQQLLENLEHMFNQINNHLQEGEGTVHHVEKNVDKLLKQFQNLVHR
NMB 19	MYGKLNHLLDELQEMFHKMNKNWHGSSDNLHDMEEQFKHMIHDLHDFMQGE<br>NHRGKLQELIQQLDKLMQQLHNHLQDQSQTVHHFDKHIDQFMNHFNHLVHR
NMB 20	MYGKLNQFVEEMNHIIHHINKNWHQRQSNLHDINHKLNQLIEQLDHFMQGE<br>NRKGKLQDVFQQLNQLMKQLNNHLQRGDQTVHHVKQQLEQVLQQIHHLVHR
NMB 21	MYGKFNELIQQIEDLMKKINKNWHEKHRNLHDLNEELQQIMHQVQNFMQGN<br>QREGKLQDIMDKMQNIFDQLHNHLQRSHQTVHHLHDNFEHLLHKVHHLVHR

**NMB 22**	MYGKLNKIMQQLEDLLKQLNKNWHGGGDNLHDLEEHLKHMLDNFHNFMQGK<br>EDSGKLQDMLNNMDNLMNNVNNHLQQKQDTVHHLDKQFQNIMEDLHHLVHR
**NMB 23**	MYGKLNKLMQQIQNIVHNMNKNWHEQSKNLHDIQKQLQHVMQHIQHFMQGG<br>KNGGKLQEVLEQMDDLMEQLNNHLQSKSRTVHHVNDDVQNLLNQFHNLVHR
**NMB 24**	MYGKLNEVMNKLEHFLKNVDKNWHNKGRNLHDLEEQLKHMVDELDHFMQGN<br>GRKGKLQQIMKQIENLLNELDNHLQDDRGTVHHLQHDFQNLIDNFHHLVHR
**NMB 25**	MYGKLNQLMKHMNDLLQKLDKNWHQHEENLHDIQHQLHQMMQHMQHFMQGE<br>QDQGKLQHLIHQMQELLKNMDNHLQSNSHTVHHLDEEFQNMFNQLDNLVHR
**NMB 26**	MYGKLNDLLDHMNKLFDQLNKNWHQNSGNLHDMEHQLEQLLQQLDDFMQGN<br>RENGKLQQIMNNVKNMINQINNHLQNENWTVHHVQDDLHKIFNEFQHLVHR
**NMB 27**	MYGKLNQFLEHFEQLLQQFHKNWHERHENLHDMNHHLEELIHKMNHFMQGR<br>NNGGKLQDLIHQMQELLKNMDNHLQSNSHTVHHVNDQFNHVLNQLHHLVHR
**NMB 28**	MYGKLNNLLNHIDHIMHKIDKNWHSRGNNLHDVEEQMEKLLKELQNFMQGN<br>GERGKLQNIIQELQQVLEKFNNHLQSDGETVHHFQQDIQQLFQQFEQLVHR
**NMB 29**	MYGKLNDVLQEVHQLLHKLDKNWHNGRDNLHDLQNNIHQFPNHLENFMQGN<br>GDSGKLQHLFKQLQDLMQHMDNHLQDKRDTVHHLEHEIKQFLKQLQNLVHR
**NMB 30**	MYGKLNKMVHQLNELVEELHKNWHGHRGNLHDLKEDIQQLLNHLNDFMQGD<br>KDHGKLQQMLDDIHKMIQELNNHLQKENGTVHHLHKNLDHMFHNLENLVHR
**NMB 31**	MYGKLNNLLQKMQDMINHLNKNWHSGRDNLHDMKKDMNQLFHQMNNFMQGN<br>REHGKLQDMLKEMHKLLHQFHNHLQSSEGTVHHVEQQLNNLLHDLDNLVHR
**NMB 32**	MYGKLNHMMQKFQELFKDLHKNWHEEKDNLHDFNQKLQEFIDHLENFMQGQ<br>GRGGKLQELVKHLQKLLNEINNHLQQQEDTVHHLNKQIDKMVNHIDDLVHR
**NMB 33**	MYGKLNNLLKQVEHVVKKVNKNWHREGRNLHDLQQNLNHLLKQVEHFMQGN<br>QDRGKLQQMLNEVEELLEKLHNHLQREDRTVHHVKEEIQNLVQQLQNLVHR
**NMB 34**	MYGKLNNLIQELKELMNNLDKNWHQRGRNLHDMDENVKQLIQNLDHFMQGE<br>NRGGKLQEVLNQLNKVLKQVHNHLQSDDDTVHHIDQNVQNVFHNFKHLVHR
**NMB 35**	MYGKLNQMLNNFQNLVHEIDKNWHQQGRNLHDLHKNINQLFQDIEHFMQGG<br>HGDGKLQHMFEKLDQLIDQFNNHLQHQSNTVHHLHHQLDQFQHILNHIQHLVHR
**NMB 36**	MYGKLNNMIDNLDQLFEQFHKNWHERGENLHDIKEEVHQLFNQFHHFMQGE<br>SDKGKLQDLIEHMDNIINQFNNHLQGKENTVHHLEHKVQNIINHVDHLVHR
**NMB 37**	MYGKLNDLLDHMNKLFDQLNKNWHQNSGNLHDMEHQLEQLLQQLDDFMQGN<br>RENGKLQQIMNNVKNMINQINNHLQNENWTVHHVQDDLHKIFNEFQHLVHR
**NMB 38**	MYGKLNELIEKMQHLINELDKNWHGDQGNLHDFEDNFQQLLNQIHHFMQGK<br>ERGGKLQQLLEQLDKMLHELNNHLQRERQTVHHLEQQLQELIHHLHDLVHR
**NMB 39**	MYGKLNNLIQELKELMNNLDKNWHQRGRNLHDMDENVKQLIQNLDHFMQGE<br>NRGGKLQEVLNQLNKVLKQVHNHLQSDDDTVHHIDQNVQNVFHNFKHLVHR
**NMB 40**	MYGKLNDIINKLNKFMKQMNKNWHGRHSNLHDMKHQLENFMEQLQHFMQGD<br>NGQGKLQELMKQINKLLNLHDNHLQEEERTVHHVQEQMNQVLNQLHNLVHR
**NMB 41**	MYGKLNHLLQELKKVLEHLNKNWHDGSDNLHDLHKDLQEVVQQMHDFMQGE<br>ENSGKLQELIKQIQQMLNKFDNHLQDERDTVHHIHQNMEQIVHNIHHLVHR
**NMB 42**	MYGKLNKMMHELHQIFHKMDKNWHNNEQNLHDFKHKLHEFFQELEDFMQGE<br>GDDGKLQHLLDNMEHMIDNLNNHLQSRNQTVHHIEQNVQDFLHHLQHLVHR
**NMB 43**	MYGKLNEFMQQVDKLLKKMDKNWHGGENNLHDMEHELDKLVDDVKDFMQGK<br>EGRGKLQDLLEKLKQLIEHFDNHLQRRGGTGHHFXHDFQNIIHHFHHLVHR
**NMB 44**	MYGKLNNMLNDIEHLLNEMHKNWHGHSRNLHDMNQDIKEIMDHLHNFMQGD<br>SHEGKLQNVFQEFEKVIKQFHNHLQKRQDTVHHLQDKFKQFLHEFDNLVHR
**NMB 45**	MYGKLNKMIKNFQNMLQEFNKNWHQGEDNLHDLKKKMEQLVNDLHNFMQGG<br>EDRGKLQDVLKNMHEIMDXINNHLQSSQDTVHHFKEDLQELFKNLNHLVHR
**NMB 46**	MYGKLNHLLKKLEHIMHDINKNWHQKDENLHDIDEELEQMMKHMHHFMQGH<br>SDDGKLQELMEEIKDVIDKLNNHLQDHRQTVHHLDNQVQQMLQHLDHLVHR
**NMB 47**	MYGKLNKVLHQLKELLDQLDKNWHRRHGNLHDIEDELHQLVKHFHHFMQGH<br>KNDGKLQDMLEKLNKMLQDMNNHLQDRKQTVHHVNNEMNDLLEQLQHLVHR
**NMB 48**	MYGKLNDLVKELNQILKEINKNWHGQGSNLHDLEQQMHELLQQMDHFMQGK<br>KGGGKLQHFVKEMNELIKQMNNHLQRQERTVHHVEQDFQKLLQDLHHLVHR
**NMB 49**	MYGKLNQMLEQLHELMKHMNKNWHRRGGNLHDLEEELENILQKLHHFMQGE<br>NNEGKLQELIEQVQQLVHEIDNHLQEQRNTVHHLDEHIEQIVDNFNHLVHR
**NMB 50**	MYGKLNSMLNDIERLLNEMHKNWHGHSRNLHDVEKELHKFLKQVQNFMQGG<br>KREGKLQDLVDELEQILEHLHNHLQQKGNTVHHLNDELKKLIDDMDNLVHR
**NMB 51**	MYGKLNQFIQELQHLLHELHKNWHDKEHNLHDIHHELDQFLNEVDHFMQGE<br>SHDGKLQNILKHMNHLIDQLNNHLQKESQTVHHVQNQLKDFLNELQNLVHR
**NMB 52**	MYGKLNQMMHKMDHLLQQLNKNWHERSGNLHDIHDKLDNLVEELKHFMQGK<br>EHHGKLQDLLNQLENIMHKLNNHLQHKKSTVHHVQNELEKMLQHLNNLVHR

Binding data for selected proteins

Table S2. Binding screen for 52 well expressed Naïve Metal Binding (NMB) Proteins. The amount of *de novo* protein that remained bound to the metalated bead after stringent washing was compared to the amount initially loaded. Proteins were grouped into three classes: If 0-33% of the protein remained bound to the bead, the protein was designated a weak binder (+); if 33-66% of the protein remained, it was classified as a moderate metal binder (++); and if 66-100% remained bound, it was classified as a strong binder (+++).

Protein	Co(II)	Cu(II)	Zn(II)
S-824	+	+++	++
S-824-HC	+	-	-
S-824-HZ	-	-	-
NMB 1	+++	++	+
NMB 2	++	+	++
NMB 3	++	+++	++
NMB 4	+++	++	+
NMB 5	+	+	++
NMB 6	++	+++	+
NMB 7	+	++	+
NMB 8	+	++	+
NMB 9	++	++	+
NMB 10	+	++	+
NMB 11	+	+	++
NMB 12	+	+++	+
NMB 13	+	++	++
NMB 14	++	++	+
NMB 15	+	+++	-
NMB 16	+	++	+
NMB 17	+	+++	+
NMB 18	+	++	+
NMB 19	-	++	+
NMB 20	+++	+++	+
NMB 21	+	+++	-
NMB 22	+	-	+
NMB 23	+	++	+
NMB 24	+	++	+
NMB 25	-	++	-

Protein	Co(II)	Cu(II)	Zn(II)
NMB 26	+	++	+
NMB 27	+	++	+
NMB 28	-	+	+
NMB 29	+	++	++
NMB 30	+	++	+
NMB 31	+	+	++
NMB 32	+	++	++
NMB 33	+	+	++
NMB 34	+	++	++
NMB 35	++	+++	+
NMB 36	-	++	+
NMB 37	+	+++	+
NMB 38	-	+++	+
NMB 39	-	++	++
NMB 40	+	++	+
NMB 41	+	++	++
NMB 42	-	+++	+
NMB 43	-	++	++
NMB 44	-	++	+
NMB 45	+	++	+
NMB 46	++	++	+
NMB 47	+	++	+
NMB 48	++	++	++
NMB 49	+	+	+
NMB 50	+	++	+
NMB 51	++	++	+
NMB 52	+	++	+

Table S3. Summary of ITC binding information from Table 1. This is compared with the binding strength estimated from binding to the metalated beads. All binding curves are shown in Figure S2.

Protein	Metal	ITC					Dialysis		Screen
		ΔH_{app} (kJ/mol)	$-T\Delta S_{app}$ (kJ/mol)	ΔG_{app} (kJ/mol)	N	K_d (µM)	N_{app}	$K_{d,app}$ (nM)	
S-824	Co(II)*	-7.36 ± 0.3	-36.9	-44.3	1	0.020 ± 0.007	1.5	700	+
		-2.25 ± 0.27	-35.2	-37.3	2	0.278 ± 0.01			
	Cu(II)	-13.3 ± 2.2	-18.3	-31.6	2	1.45± 0.82	1.5	700	+++
	Zn(II)	-33.5 ± 3.8	-1.75	-31.8	3	2.74 ± 0.94	3	1000	++
NMB 39	Co(II)	-47.4 ± 7.0	15.1	-31.5	1	2.17 ± 0.89	2	700	-
	Cu(II)	-19.6 ± 2.1	-13.1	-32.7	2	1.92 ± 0.82	1	600	++
	Zn(II)	-44.5 ± 3.0	11.2	-33.3	1.5	1.50 ± 0.42	4	1000	++
HisZero	Co(II)	-	-	-	-	-	-	-	-
	Cu(II)	-	-	-	-	-	-	-	-
	Zn(II)	-8.23 ± 0.67	-26.1	-34.4	1	0.961± 0.350	1	1500	-

* 20 nM is near the Limit of Detection for ITC. This is the best fit of a two-site model, but should be viewed as an estimate rather than a precise determination.

Apparent Dissociation Constant:

The dissociation constant (K_d) for one site binding one metal describes the following system at equilibrium

$$[\text{Metal}]_{\text{free}} + [\text{Receptor}]_{\text{free}} \underset{k_{-1}}{\overset{k_1}{\rightleftarrows}} [\text{Metal: Receptor}]_{\text{bound}} \tag{1}$$

With these, the K_d of a single site is defined as the ratio of dissociation rate to association rate

$$K_d = \frac{k_{-1}}{k_1} = \frac{[\text{Metal}]_{\text{free}}[\text{Receptor}]_{\text{free}}}{[\text{Metal: Receptor}]_{\text{bound}}} = \frac{[M]_f[R]_f}{[R]_B} \tag{2}$$

Which can be written in terms of bound receptor ($[R]_B$) and total receptor ($[R]_T$)

$$K_d = \frac{[M]_f\ ([R]_T - [R]_B)}{[R]_B} \tag{3}$$

Which can be rewritten as

$$\frac{[R]_B}{[R]_T} = \frac{[M]_f}{K_d + [M]_f} \tag{4}$$

Equation (5) is what is used to fit the $K_d$curve for a single binding site.

For multiple independent binding sites with somewhat overlapping affinity, as we find in our *de novo* proteins, the observed binding is a linear combination of the contributing events. We denote the apparent affinity across n similar binding sites $K_{d,app}$, and define it as the average of all component dissociation constants $K_{d,n}$.

$$K_{d,app} = \frac{K_{d,1}+K_{d,2}+\cdots K_{d,n}}{n} = \frac{\left(\frac{[M]_f\ ([R]_T-[R]_{B,1})}{[R]_{B,1}}+\frac{[M]_f\ ([R]_T-[R]_{B,2})}{[R]_{B,2}}+\cdots+\frac{[M]_f\ ([R]_T-[R]_{B,n})}{[R]_{B,n}}\right)}{n} \tag{5}$$

Because all receptors are on the same protein of concentration $[R]_T$ and total metal is in equilibrium across the system, the only additional variable is the metal bound to each receptor $[R]_{B,n}$. This can be rewritten to mirror equation (4)

$$\frac{1}{n}\left(\frac{[M]_f[R]_T}{[R]_{B,1}} + \frac{[M]_f[R]_T}{[R]_{B,2}} + \cdots + \frac{[M]_f[R]_T}{[R]_{B,n}}\right) = K_{d,app} + [M]_f \tag{6}$$

$$\frac{[R]_{B,1} + [R]_{B,2} + \cdots + [R]_{B,n}}{[R]_T} = \frac{[M]_f}{K_{d,app} + [M]_f} \tag{7}$$

$$\frac{[R]_{B,app}}{[R]_T} = \frac{[M]_f}{K_{d,app} + [M]_f} \tag{8}$$

$[R]_{B,app}$ is the bound concentration measured in the experiment, and is the total bound across all sites. Thus, the left-hand side of equation (8) is the "Bound Equivalents" y-axis in all equilibrium dialysis plots. Equation (8) is what is used to fit the $K_{D,app}$ curve for all *de novo* proteins.

Binding curves for selected proteins

Figure S1: Binding curves for all proteins characterized by equilibrium dialysis with Co^{2+}, Cu^{2+}, and Zn^{2+}.

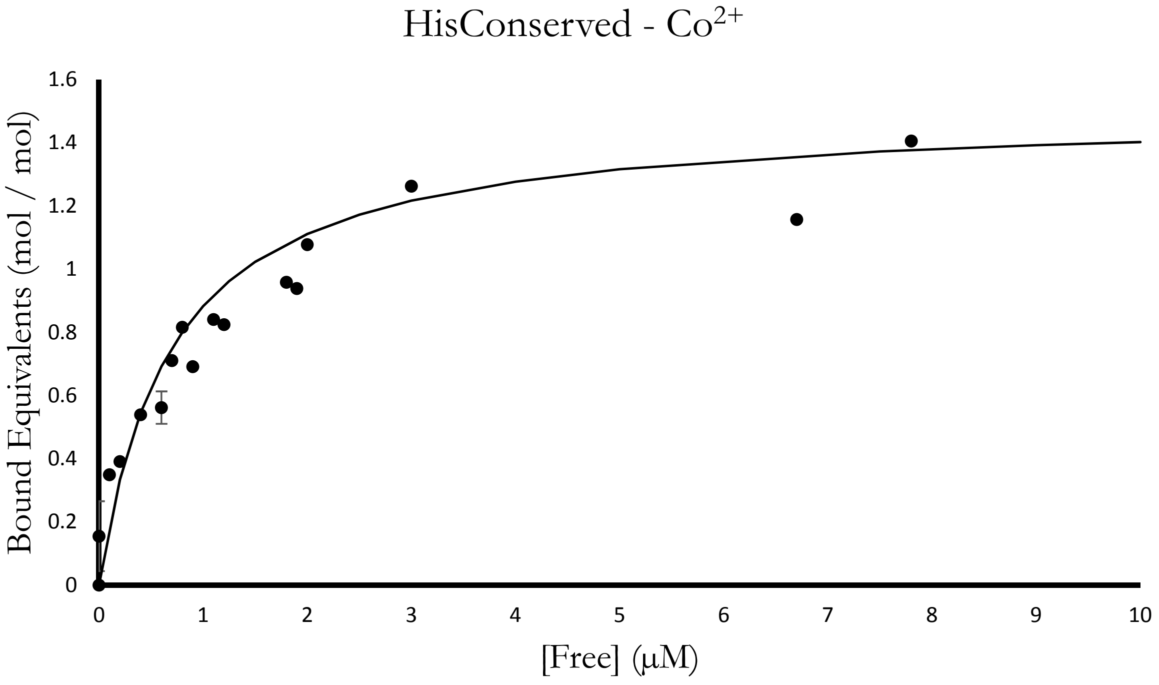

Kd,app (nM)	900	Max Equivalents (mol/mol)	1.5

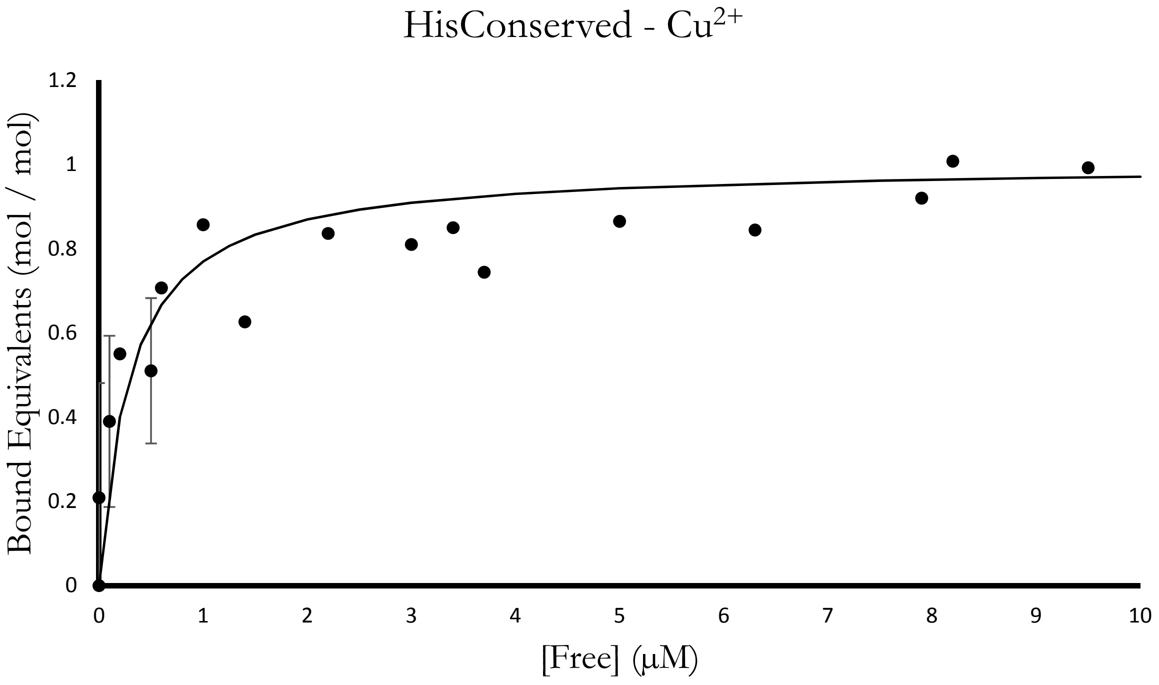

$K_{d,app}$ (nM)	300	Max Equivalents (mol/mol)	1

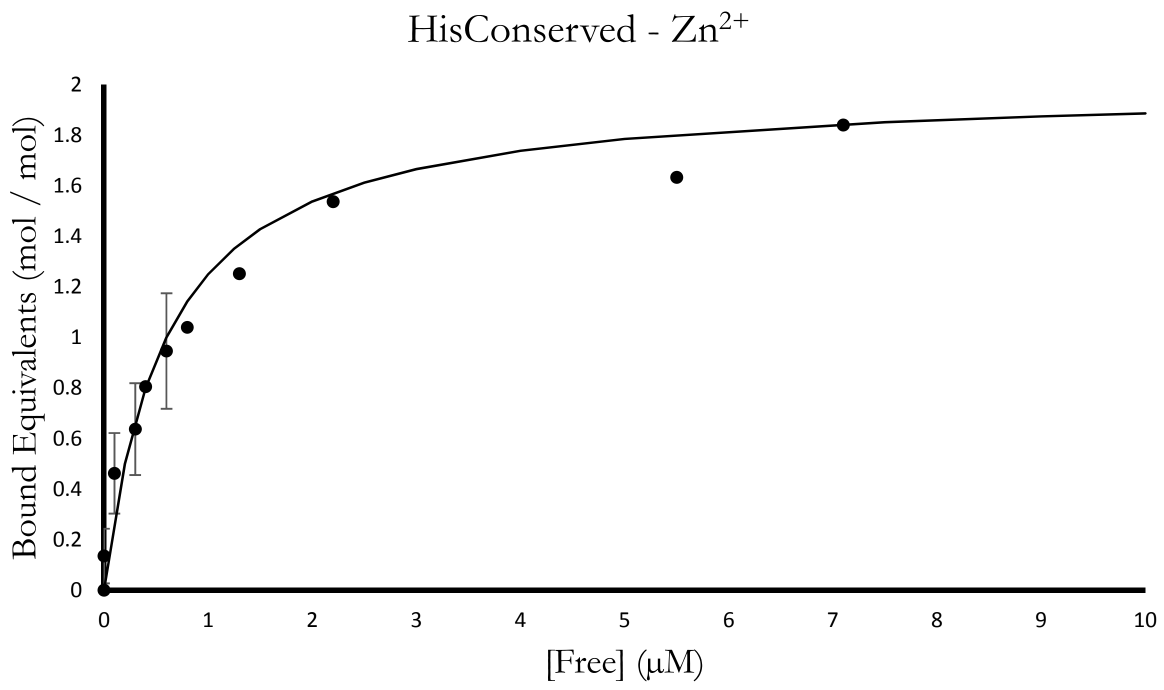

$K_{d,app}$ (nM)	600	Max Equivalents (mol/mol)	2

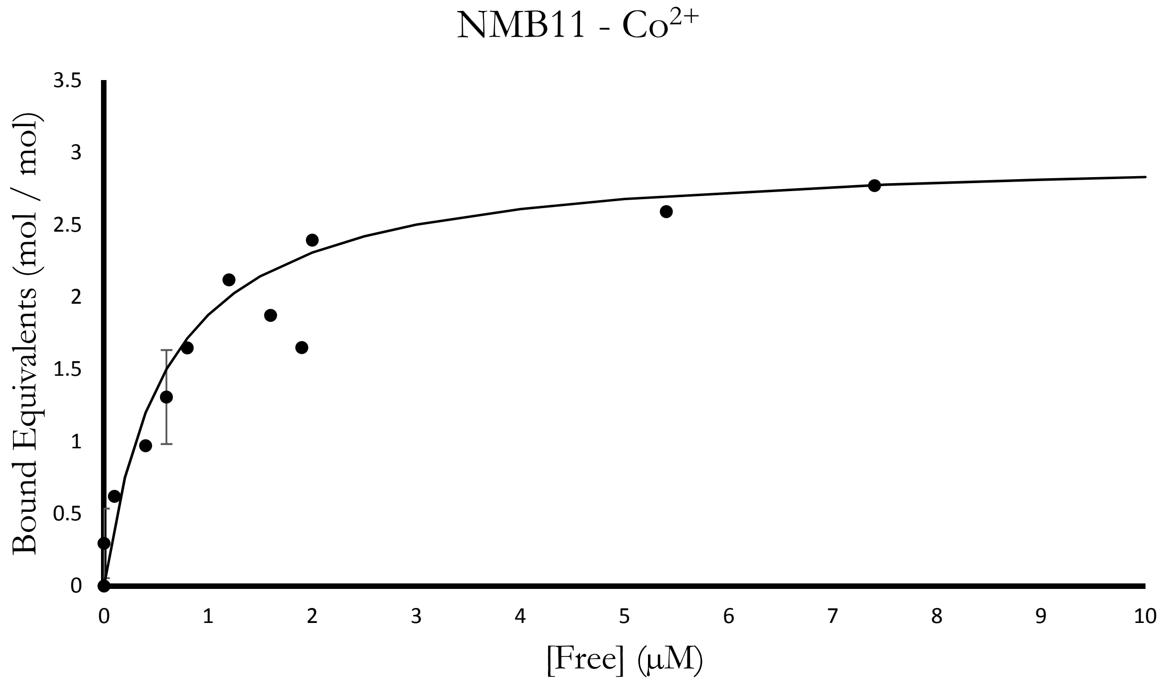

$K_{d,app}$ (nM)	600	Max Equivalents (mol/mol)	3

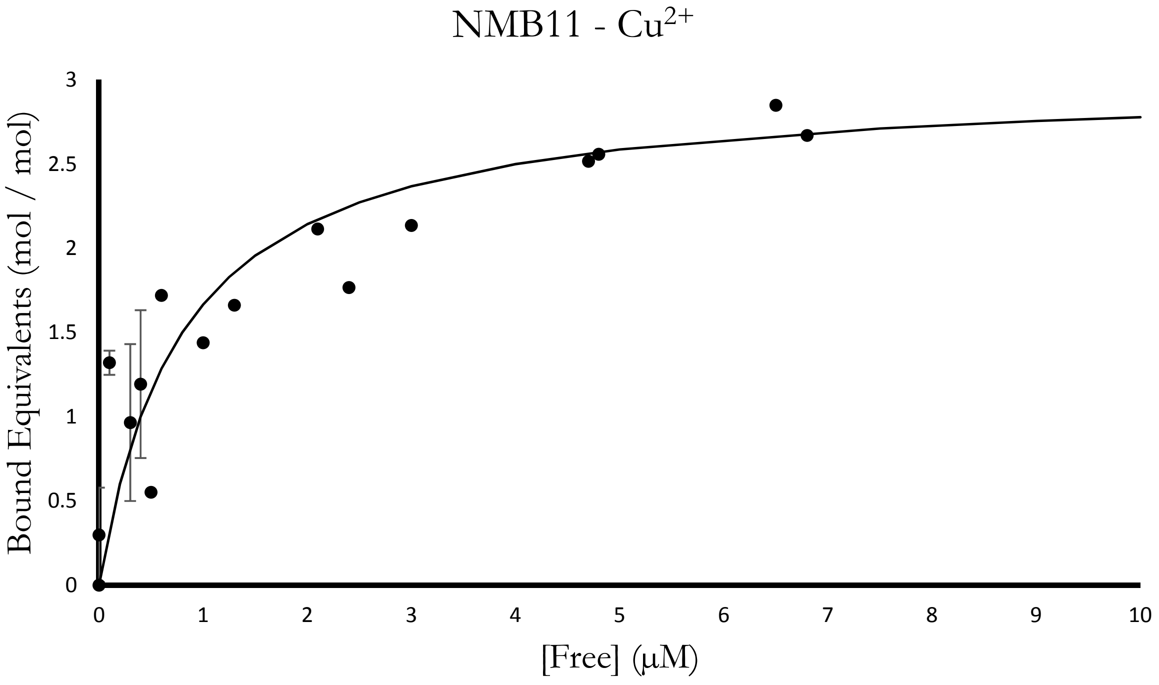

$K_{d,app}$ (nM)	800	Max Equivalents (mol/mol)	3

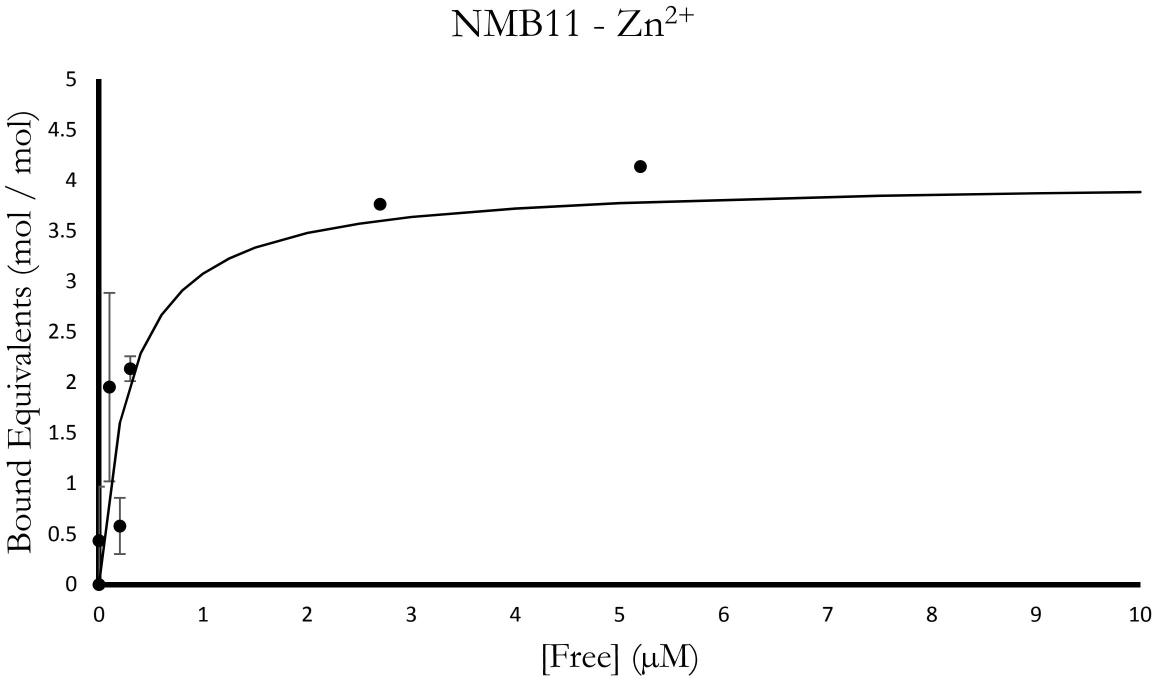

$K_{d,app}$ (nM)	300	Max Equivalents (mol/mol)	4

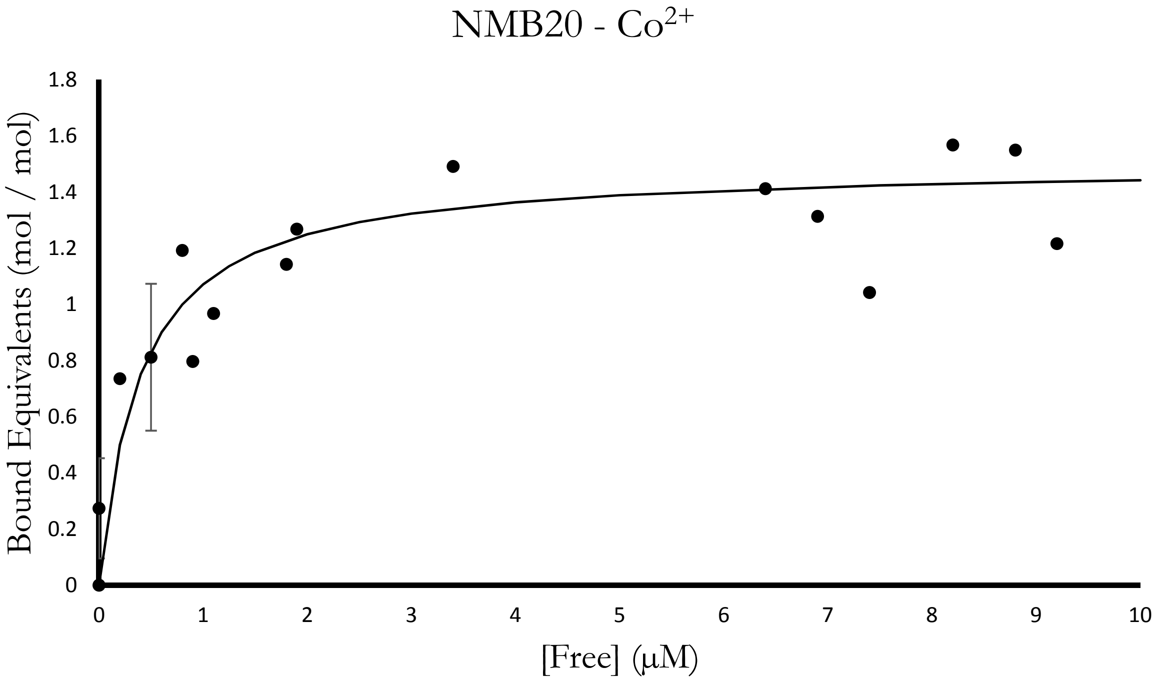

$K_{d,app}$ (nM)	300	Max Equivalents (mol/mol)	1.5

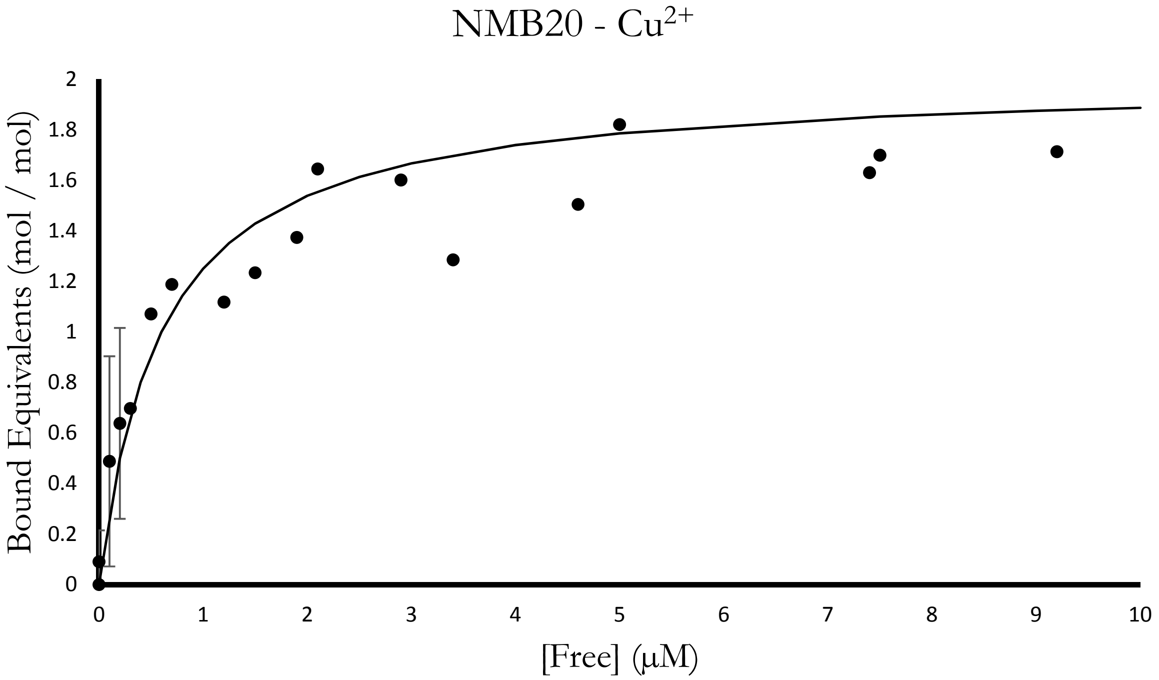

$K_{d,app}$ (nM)	600	Max Equivalents (mol/mol)	2

NMB20 - Zn^{2+}

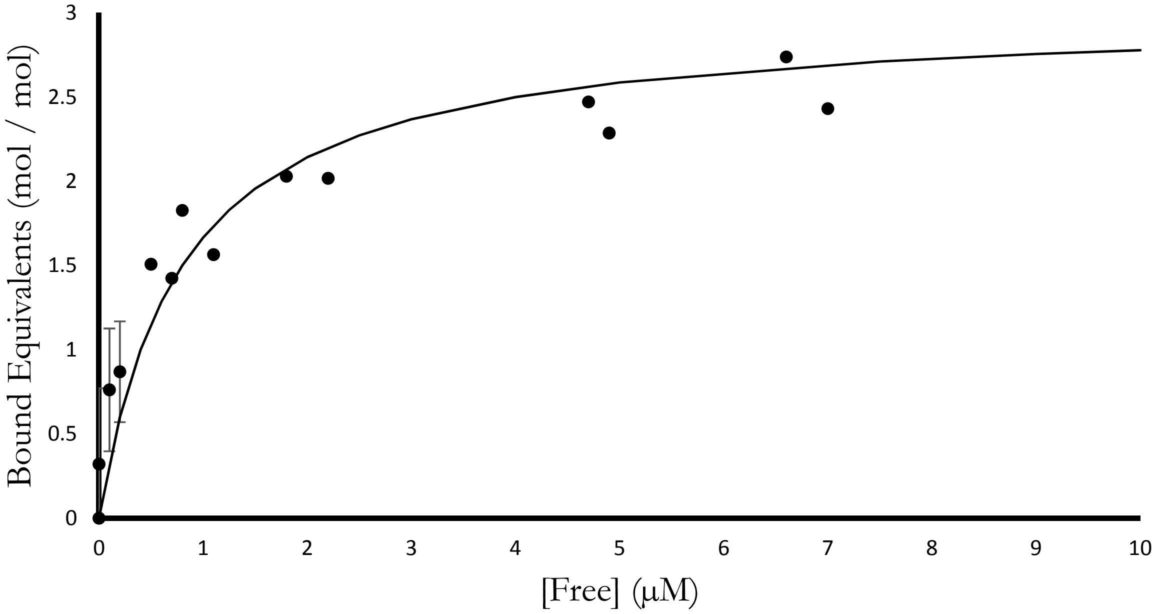

$K_{d,app}$ (nM)	800	Max Equivalents (mol/mol)	3

NMB24 - Co^{2+}

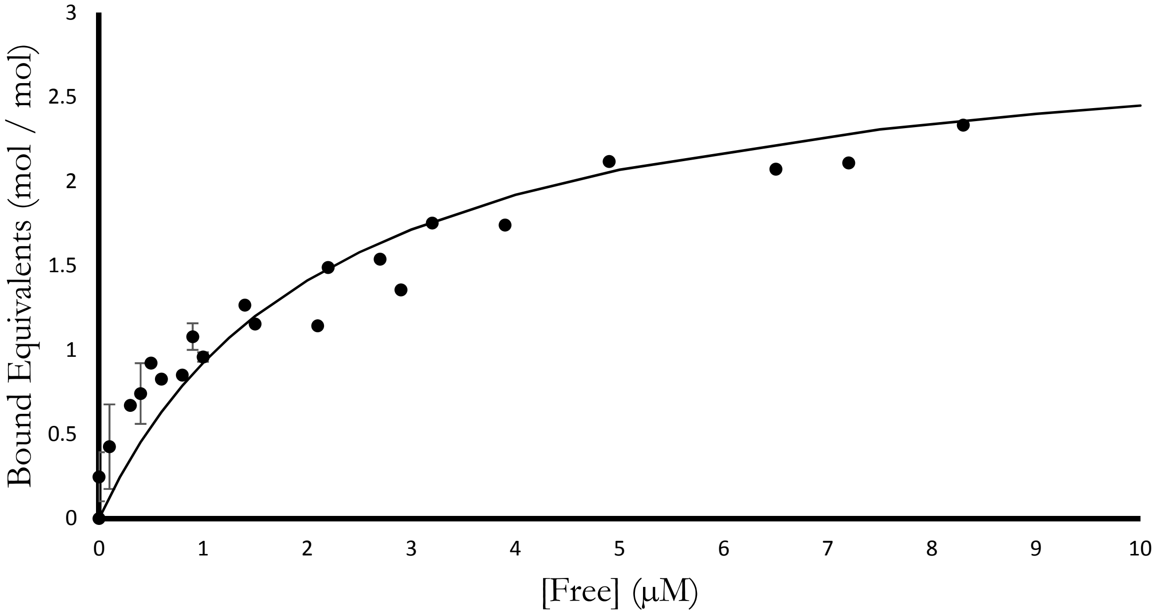

$K_{d,app}$ (nM)	2300	Max Equivalents (mol/mol)	3

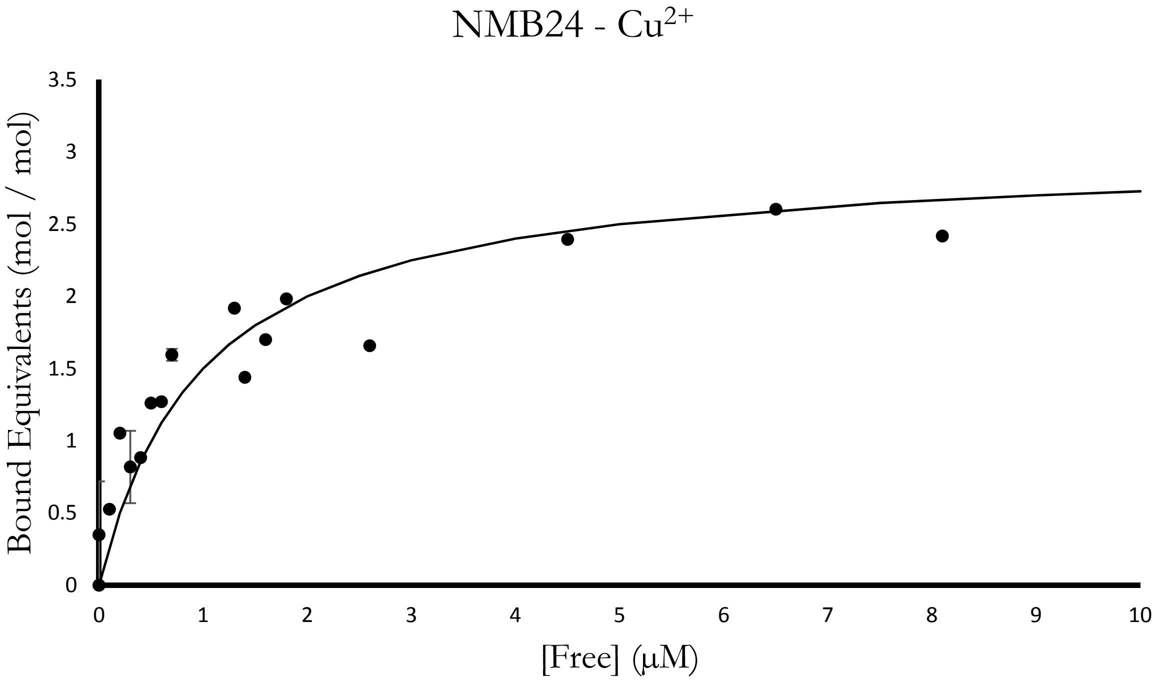

Kd,app (nM)	1000	Max Equivalents (mol/mol)	3

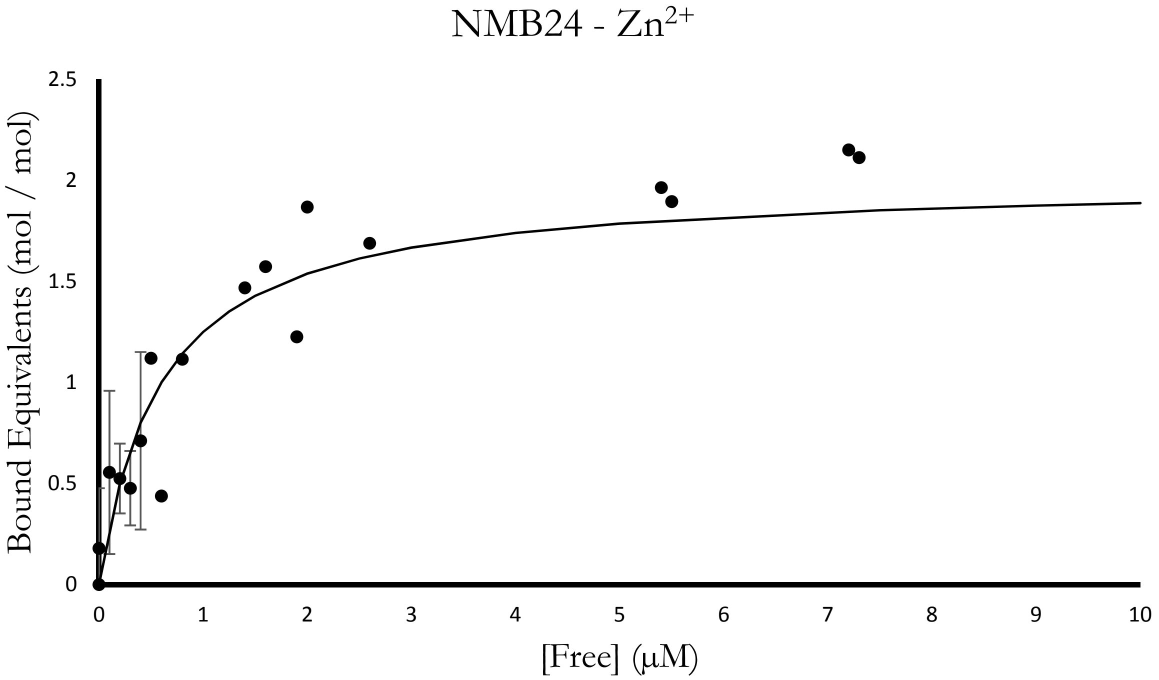

Kd,app (nM)	600	Max Equivalents (mol/mol)	2

NMB25 - Co^{2+}

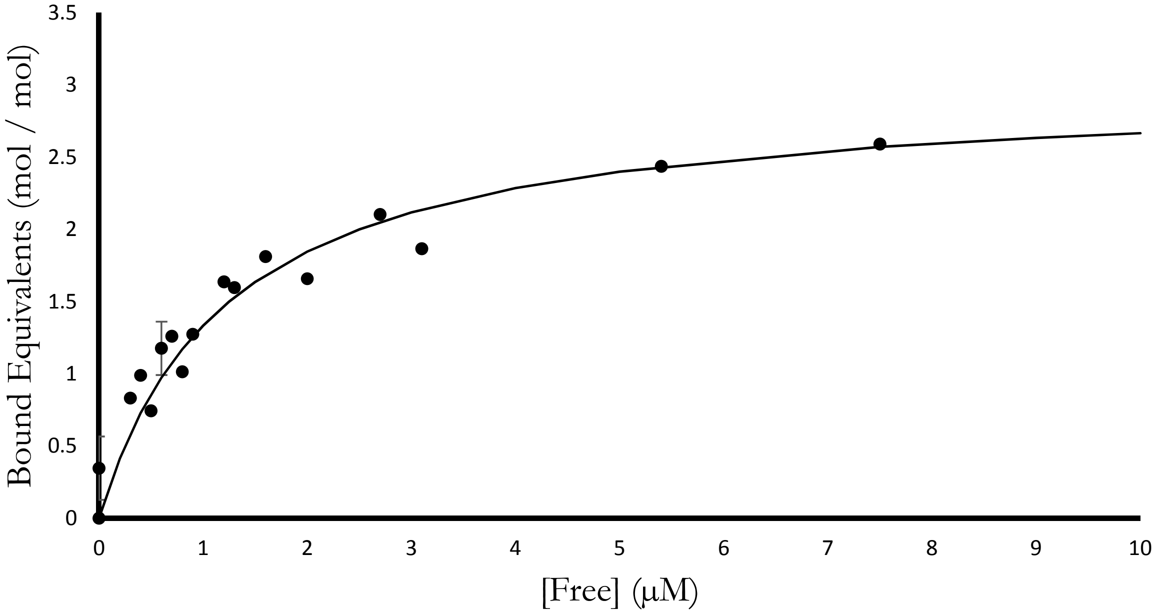

$K_{d,app}$ (nM)	1200	Max Equivalents (mol/mol)	3

NMB25 - Cu^{2+}

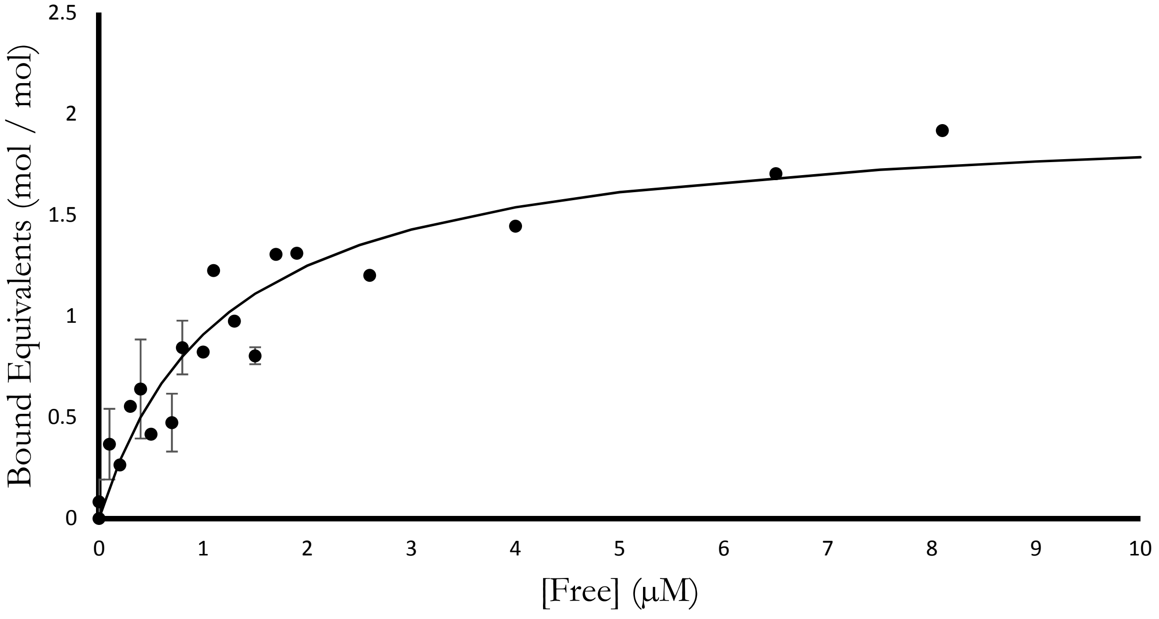

$K_{d,app}$ (nM)	1200	Max Equivalents (mol/mol)	2

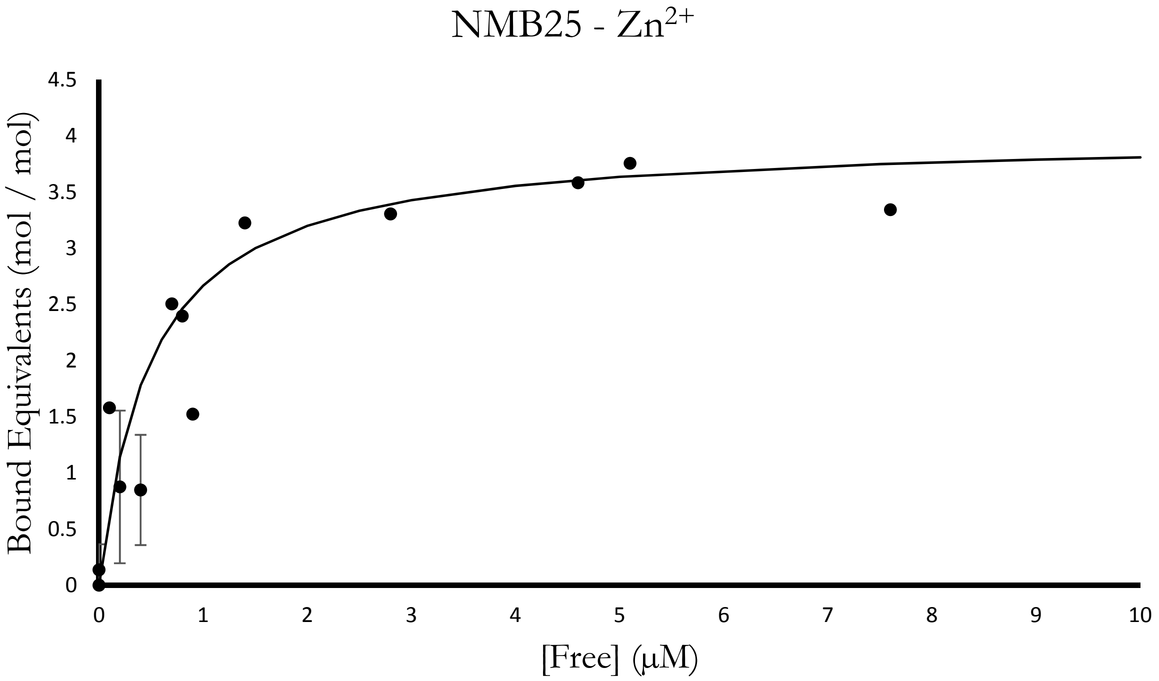

$K_{d,app}$ (nM)	500	Max Equivalents (mol/mol)	4

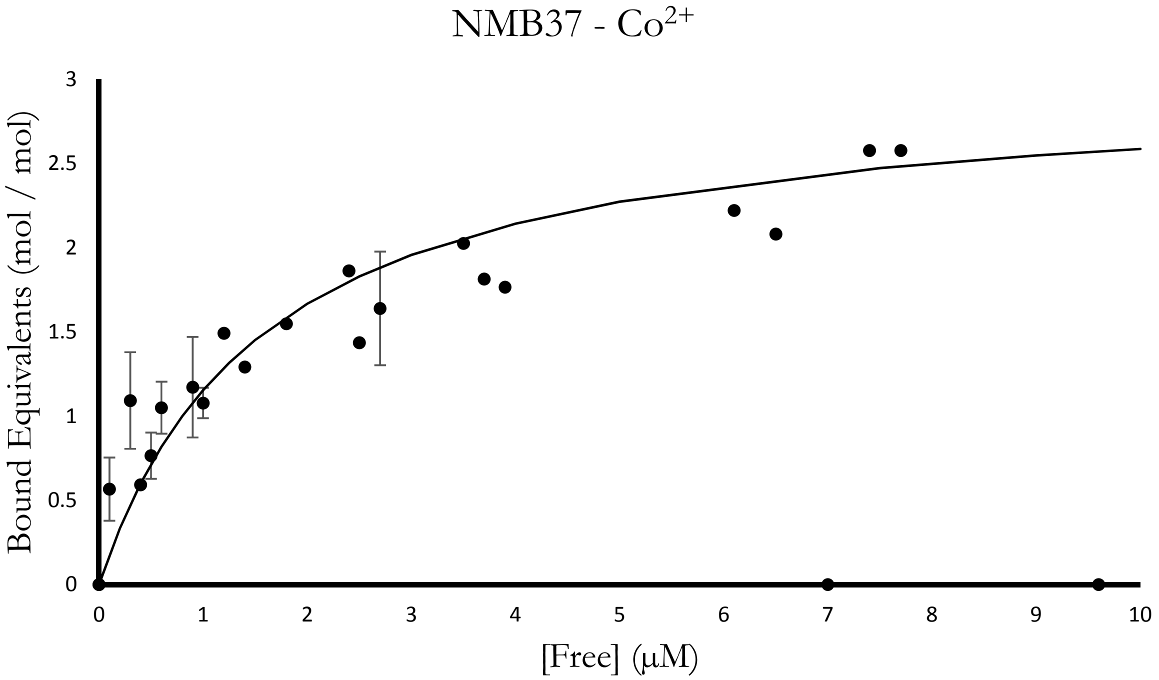

$K_{d,app}$ (nM)	1600	Max Equivalents (mol/mol)	3

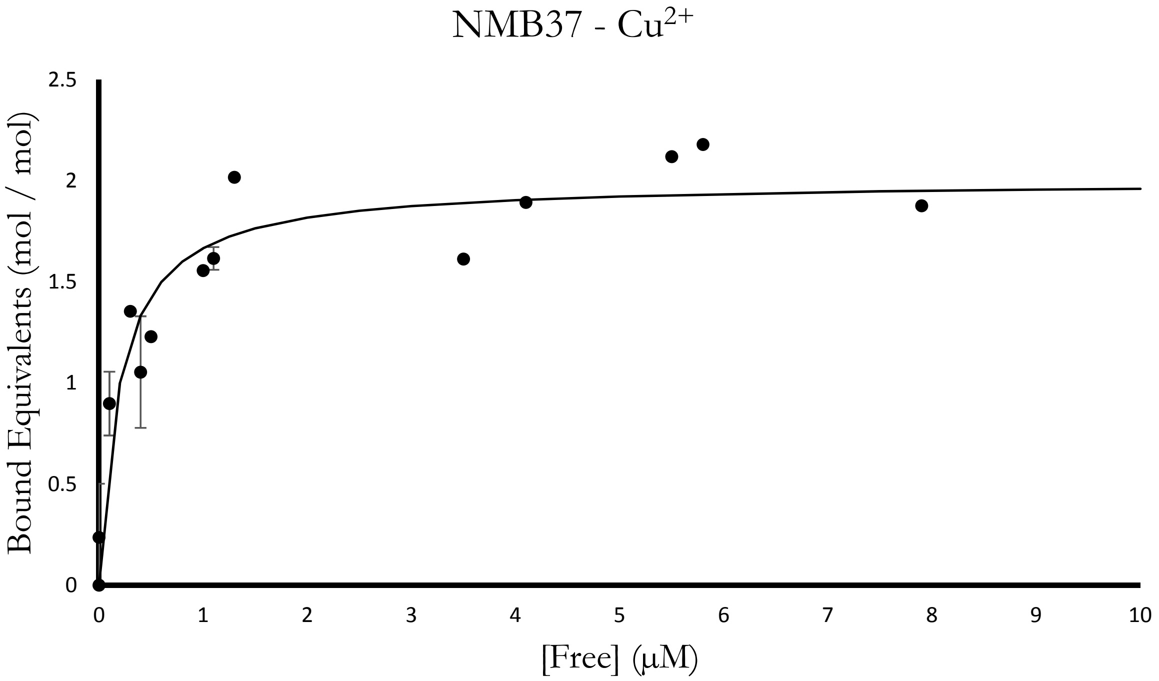

$K_{d,app}$ (nM)	200	Max Equivalents (mol/mol)	2

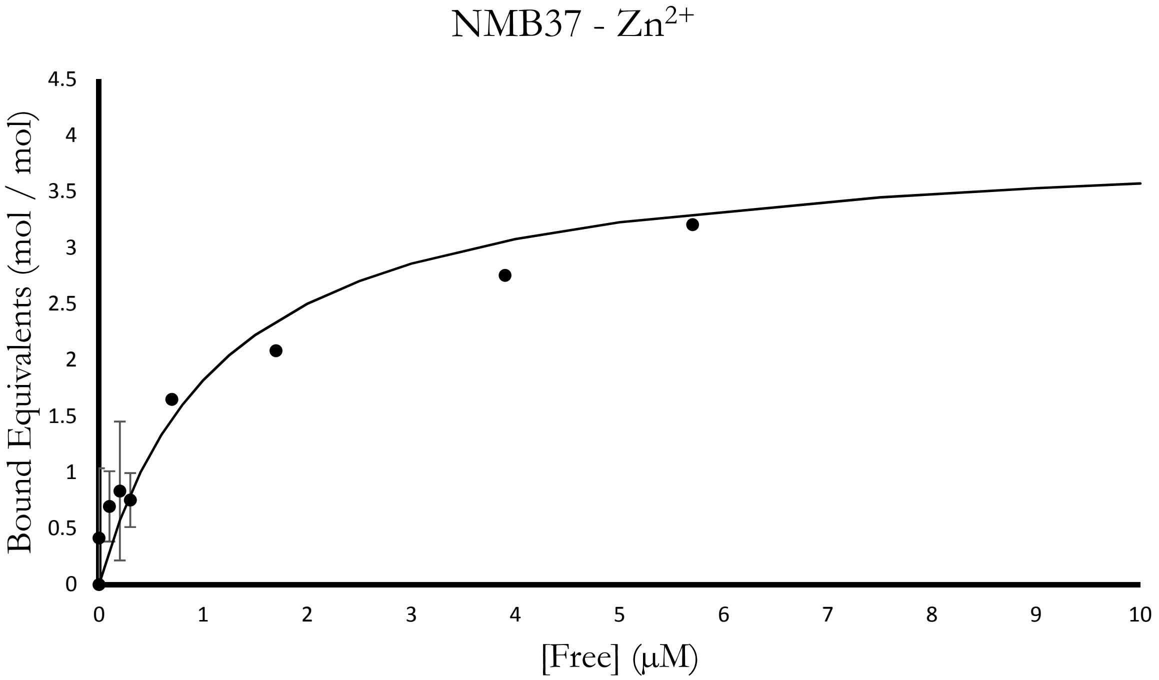

$K_{d,app}$ (nM)	1200	Max Equivalents (mol/mol)	4

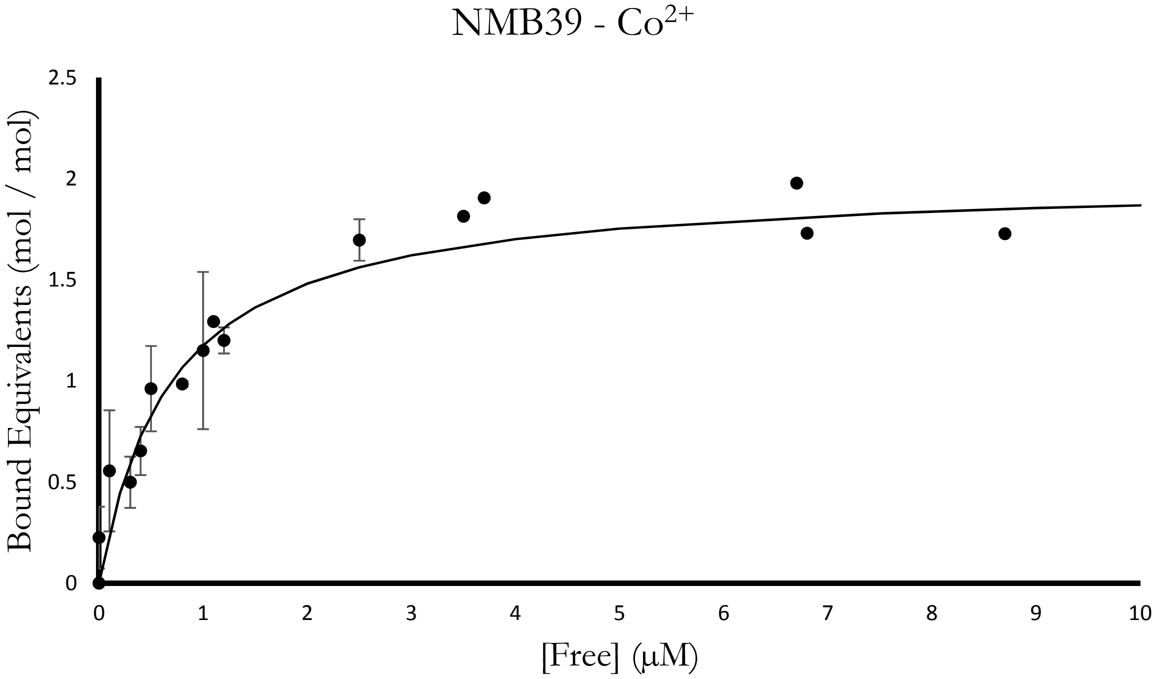

Kd,app (nM)	700	Max Equivalents (mol/mol)	2

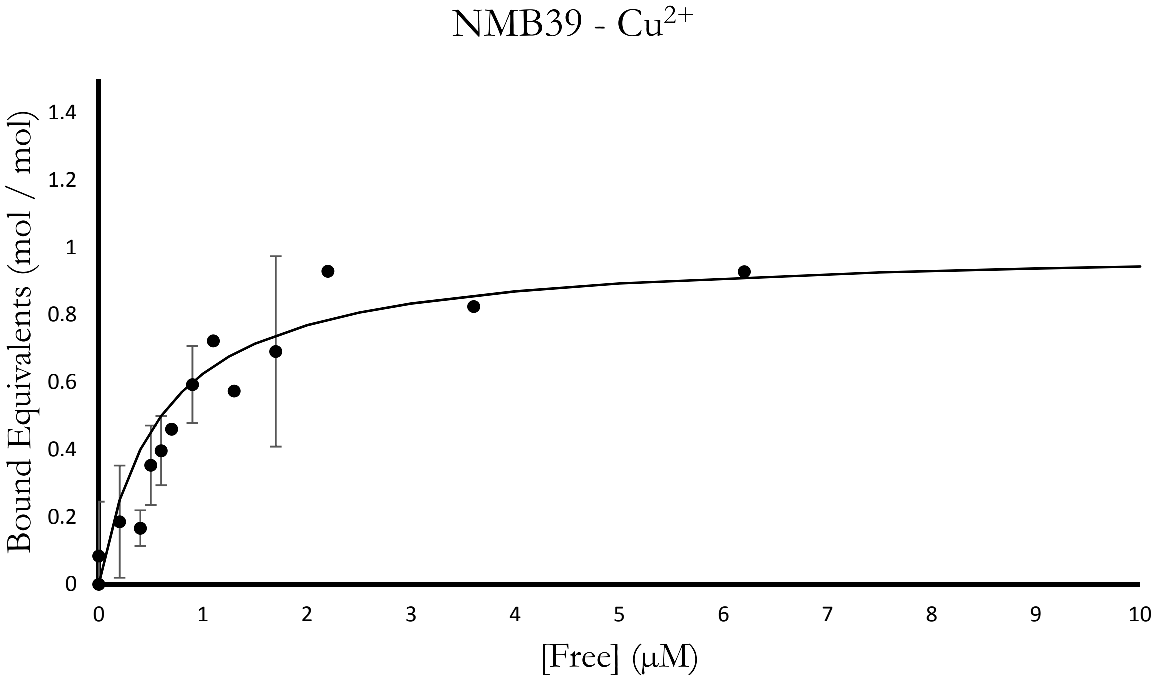

Kd,app (nM)	600	Max Equivalents (mol/mol)	1

NMB39 - Zn^{2+}

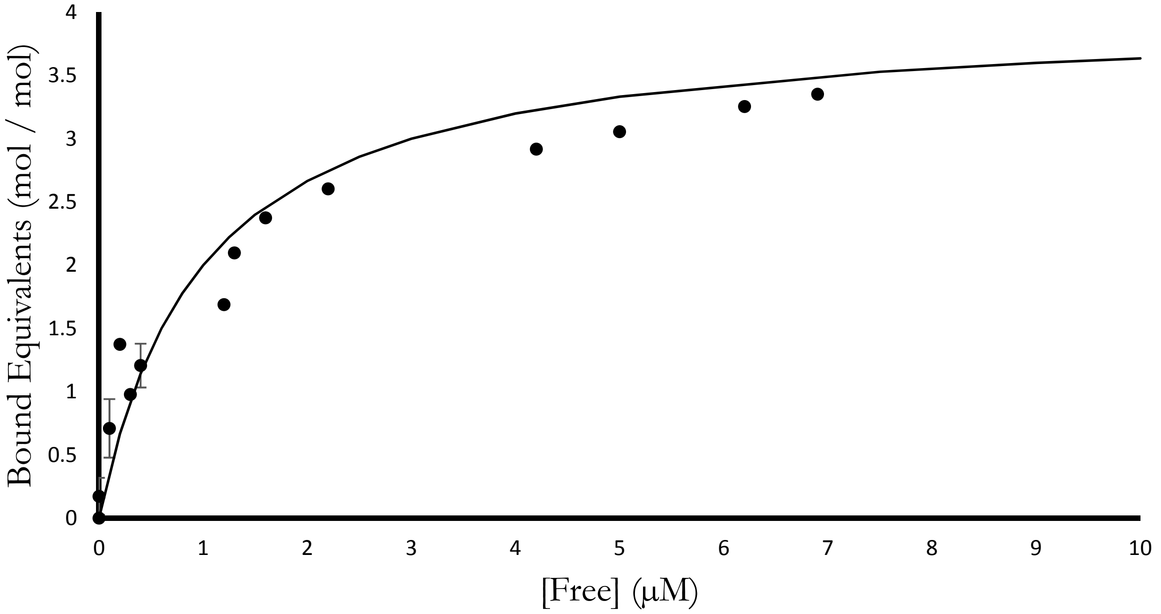

**$K_{d,app}$ (nM)**	**1000**	**Max Equivalents (mol/mol)**	**4**

S824 - Co^{2+}

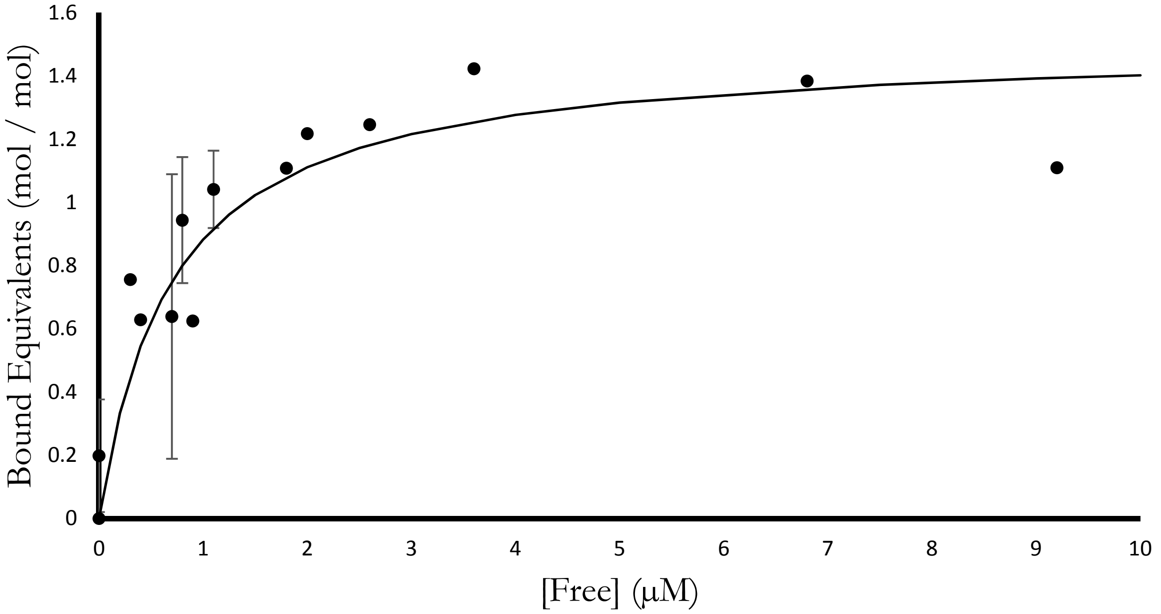

**$K_{d,app}$ (nM)**	**700**	**Max Equivalents (mol/mol)**	**1.5**

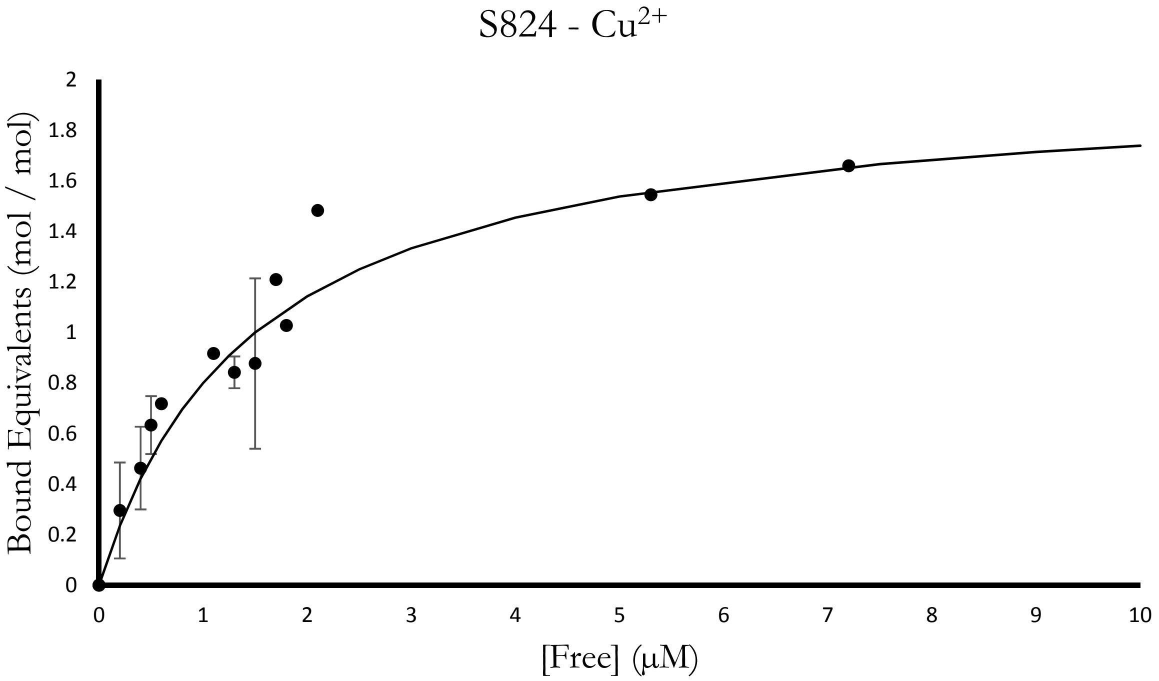

$K_{d,app}$ (nM)	700	Max Equivalents (mol/mol)	1.5

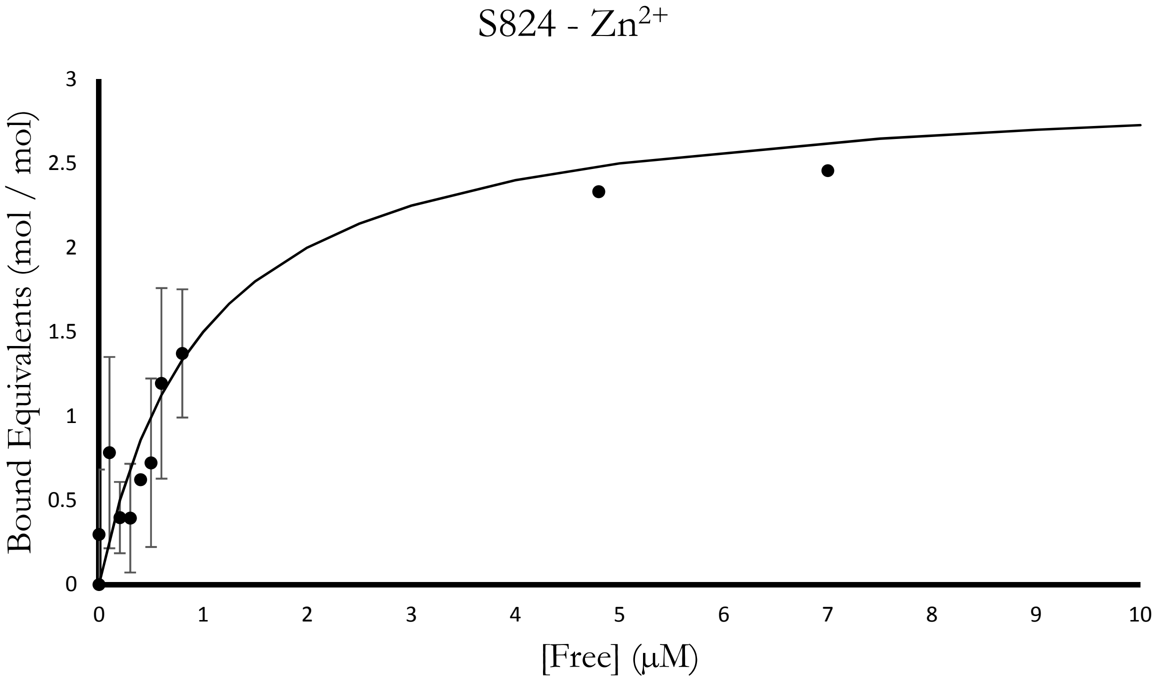

$K_{d,app}$ (nM)	1000	Max Equivalents (mol/mol)	3

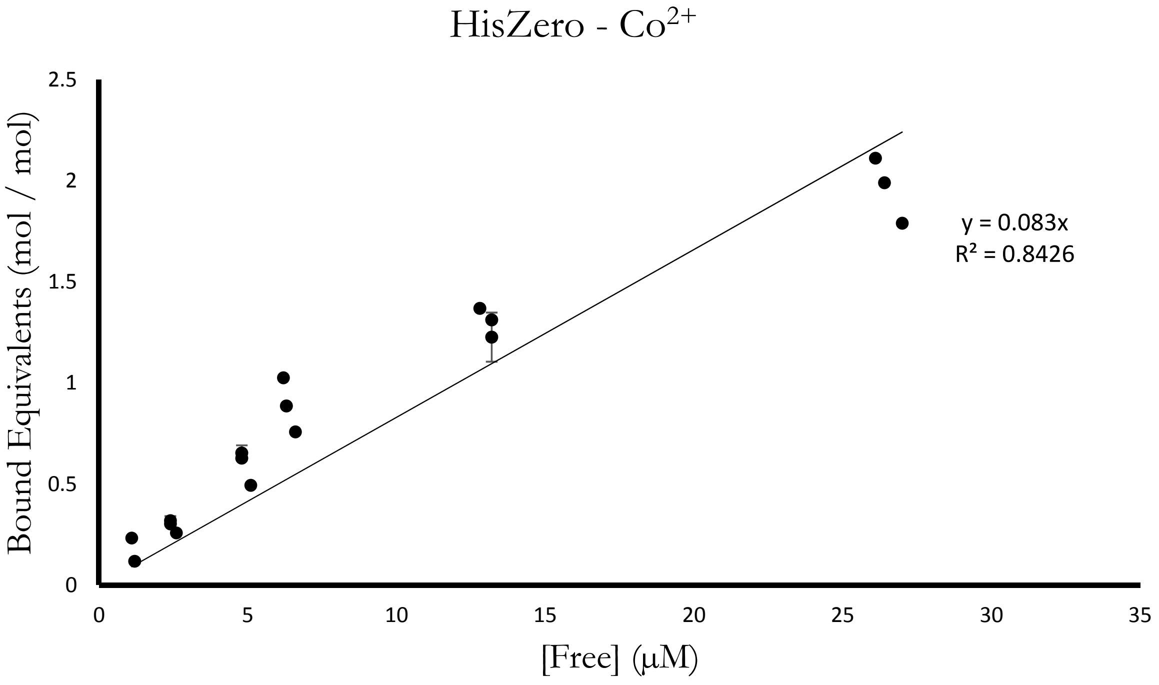

Binding is proportional to Free metal - nonspecific

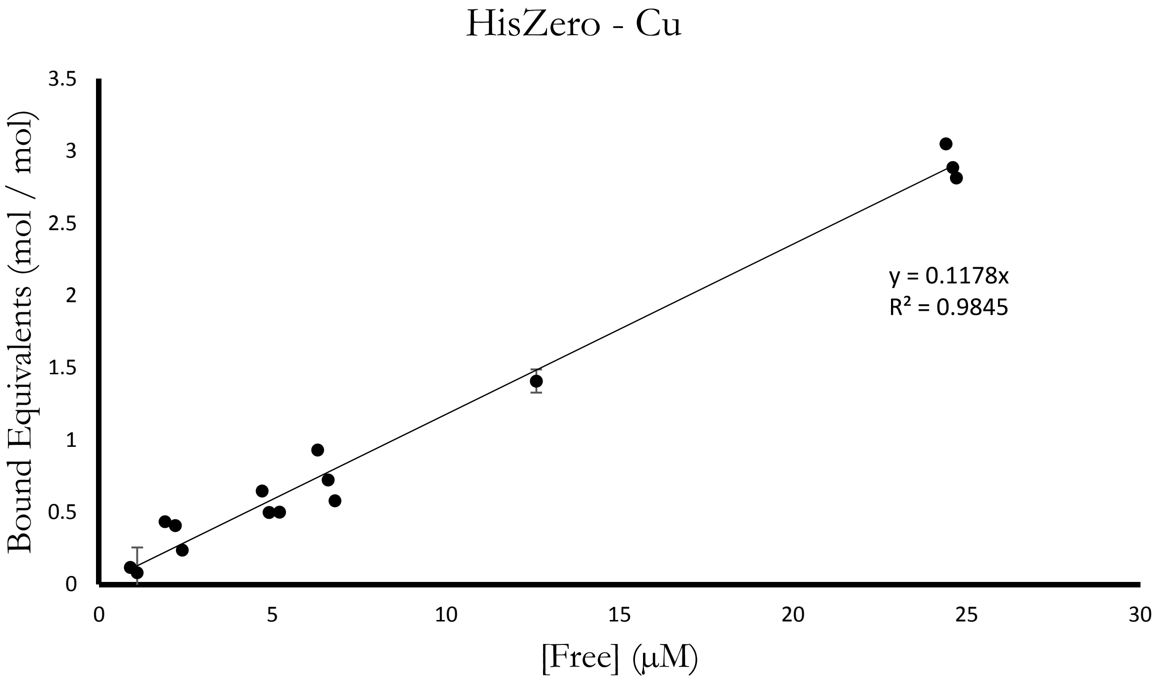

Binding is proportional to Free metal - nonspecific

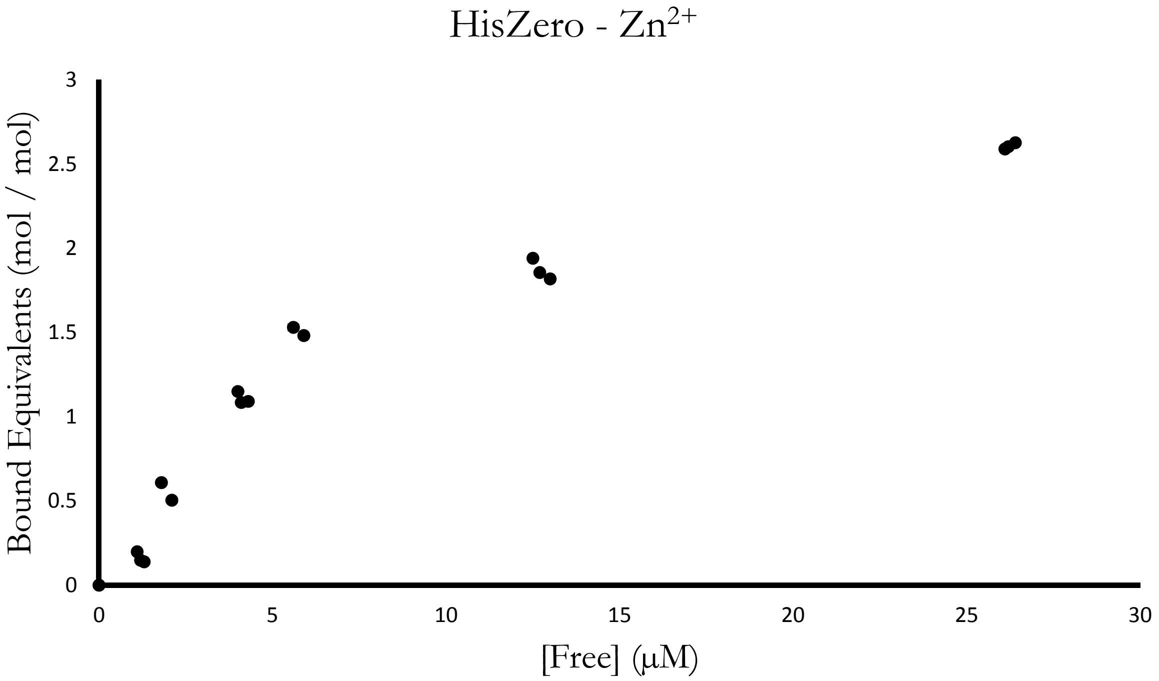

Above 5μM free zinc, the nonspecific binding is apparent:

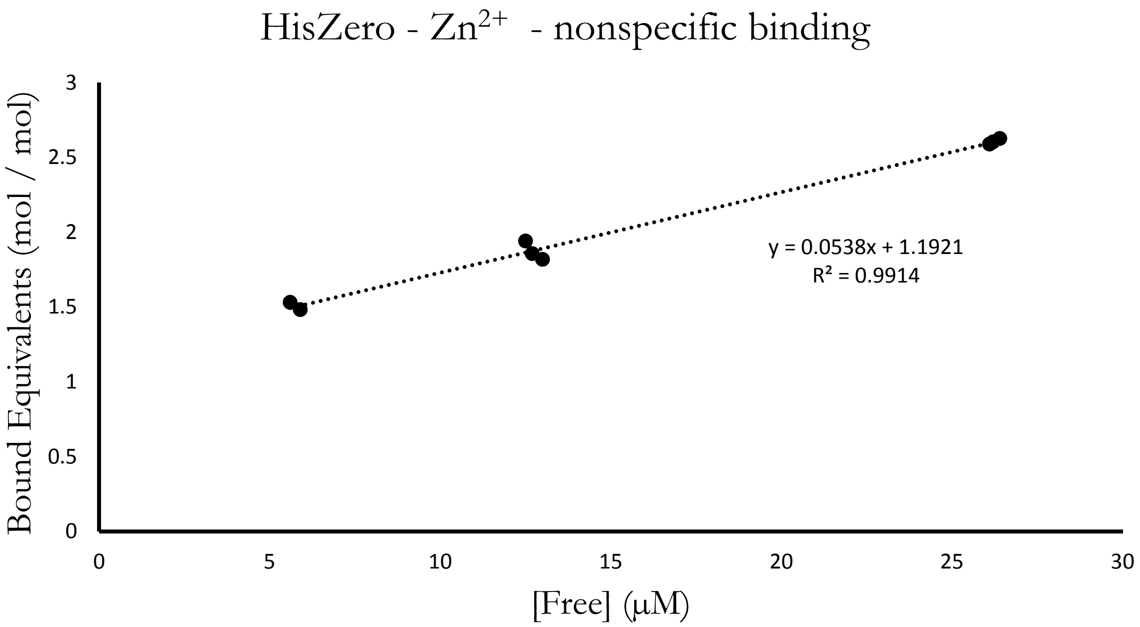

Accounting for nonspecific binding gives the following curve:

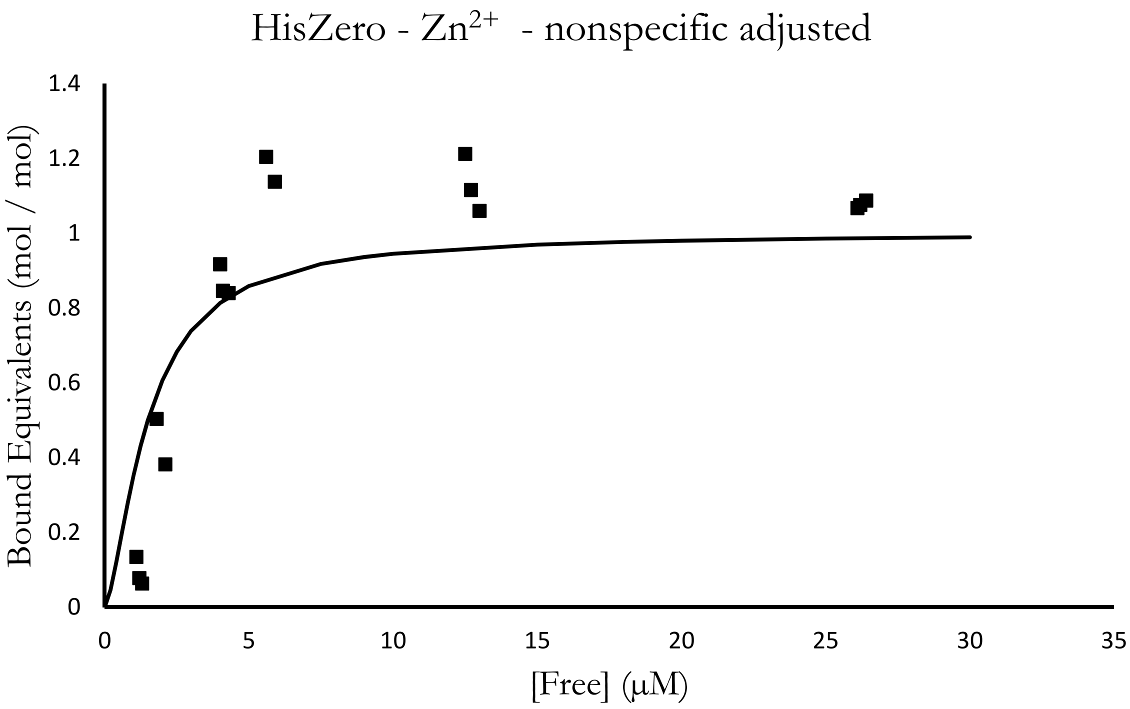

$K_{d,app}$ (nM)	1500	Max Equivalents (mol/mol)	1

Figure S2: Binding curves for all proteins characterized by ITC with Co^{2+}, Cu^{2+}, and Zn^{2+}. Raw data is presented at the top while the fit curve is below. Tabluated below each figure is the protein and metal concentrations used, and the determined affinity and thermodynamic terms, or the raw enthalpy of dilution for nonbinding events. Noise in S-824 binding copper is due to metal-mediated precipitation.

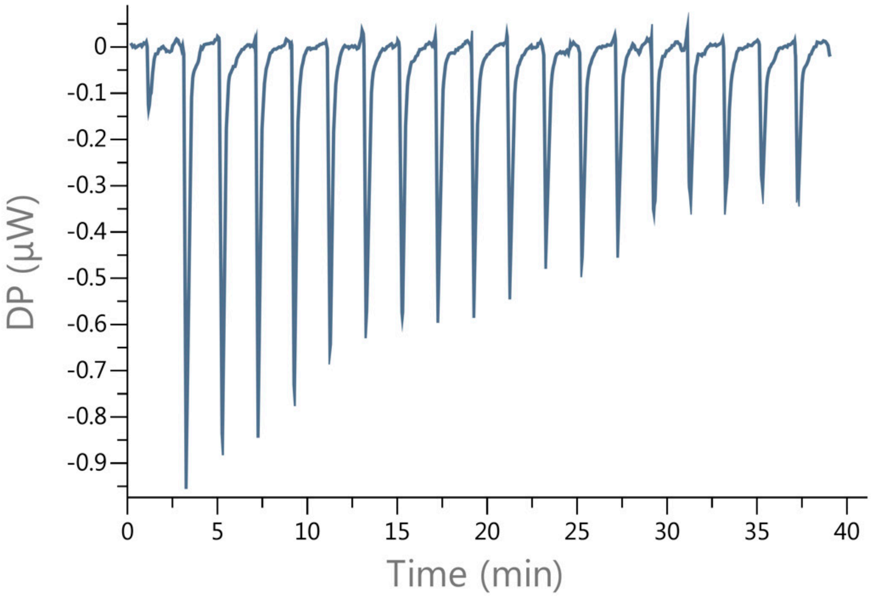

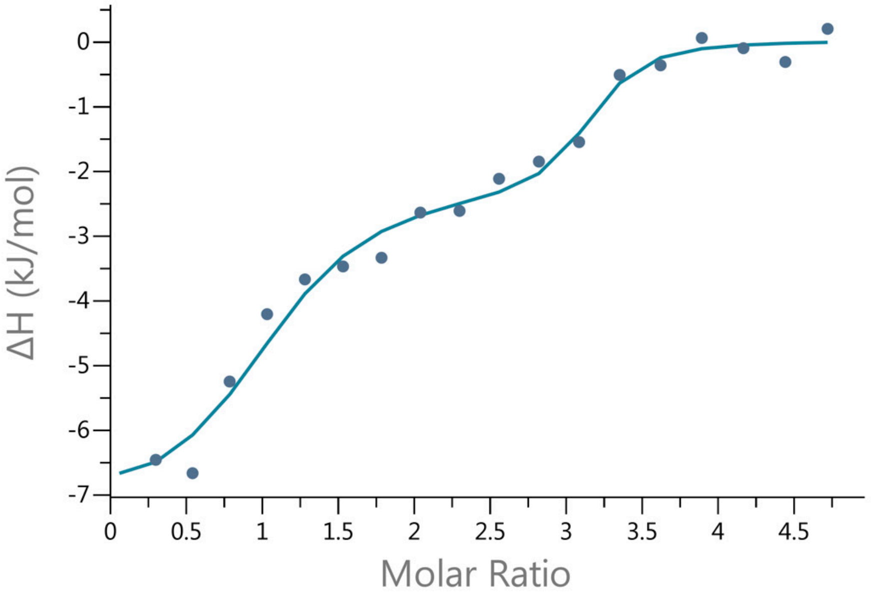

**Protein:** 20μM S-824		**Metal:** 500μM Co(II)
**N_1:** 0.94 ± 0.03		**$K_{D,1}$:** 20 ± 7 nM *
**ΔH_1:** -7.36 ± 0.3 kJ/mol	**$-T\Delta S_1$:** -36.9 kJ/mol	**ΔG_1:** -44.3 kJ/mol
**N_2:** 2.11 ± 0.06		**$K_{D,2}$:** 0.278 ± 0.01 μM
**ΔH_2:** -2.25 ± 0.27 kJ/mol	**$-T\Delta S_2$:** -35.2 kJ/mol	**ΔG_2:** -37.3 kJ/mol

* 20 nM is near the Limit of Detection for ITC. This is the best fit of a two-site model, but should be viewed as an estimate rather than a precise determination. .

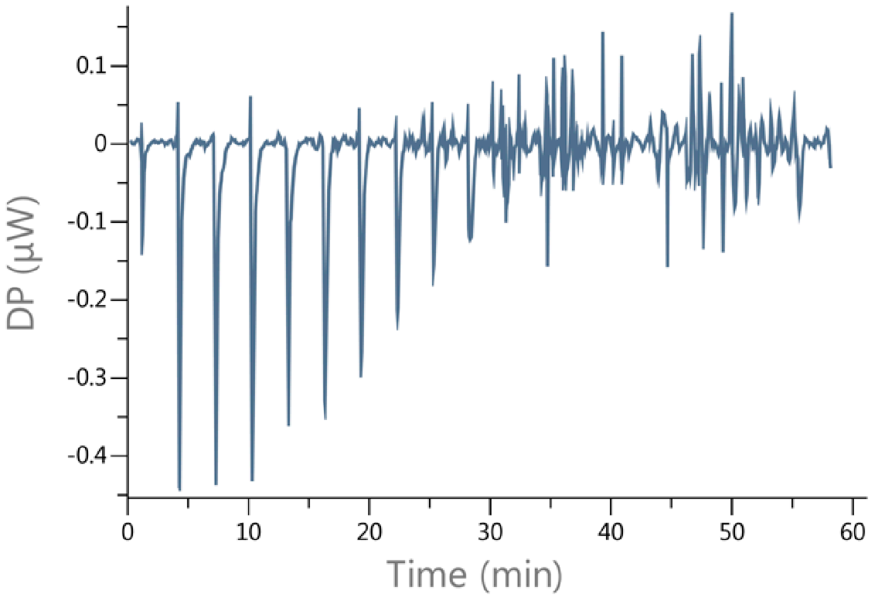

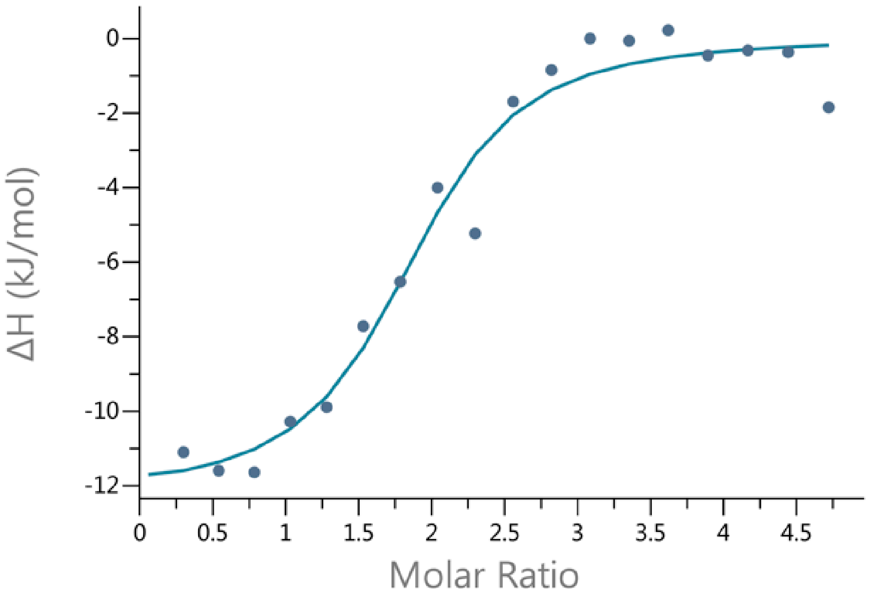

ITC noise at t > 29min is the metal-induced precipitation of the protein.

**Protein:** 10µM S-824		**Metal:** 250µM Cu(II)
**N:** 1.81 ± 0.09		**K_D:** 0.853± 0.442 µM
**ΔH:** -12.3 ± 1.2 kJ/mol	**-TΔS:** -22.4 kJ/mol	**ΔG:** -34.7 kJ/mol

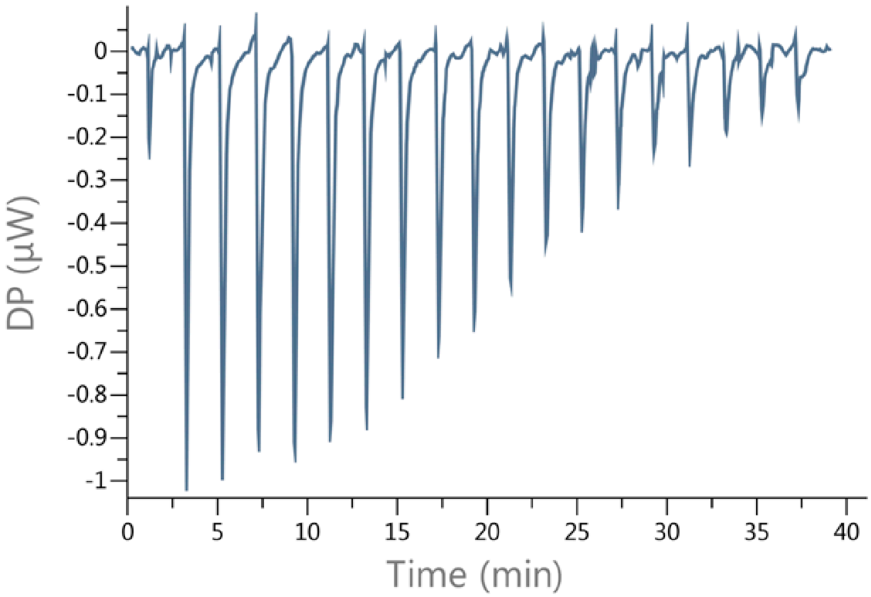

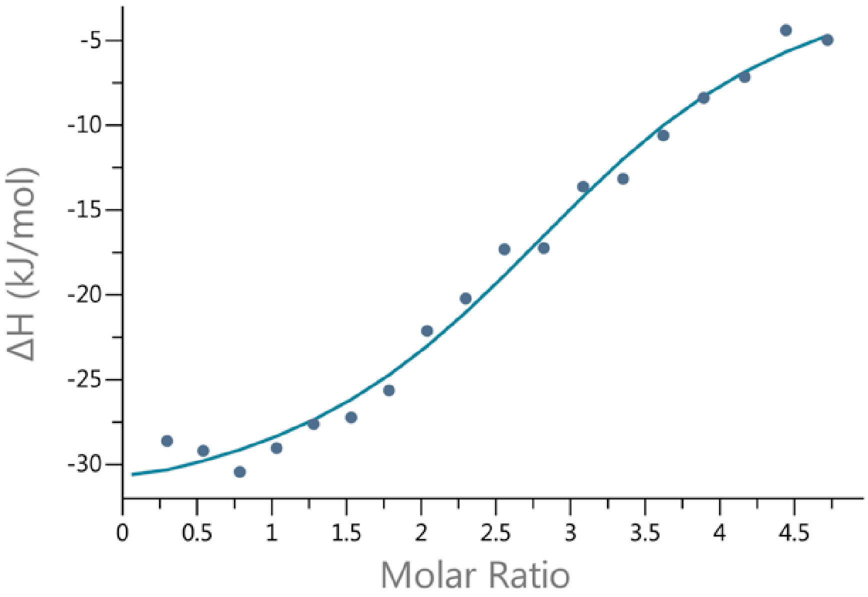

**Protein:** 10μM S-824		**Metal:** 250μM Zn(II)
**N:** 3.02 ± 0.20		**K_D:** 2.74 ± 0.94 μM
**ΔH:** -33.5 ± 3.8 kJ/mol	**-TΔS:** 1.75 kJ/mol	**ΔG:** -31.8 kJ/mol

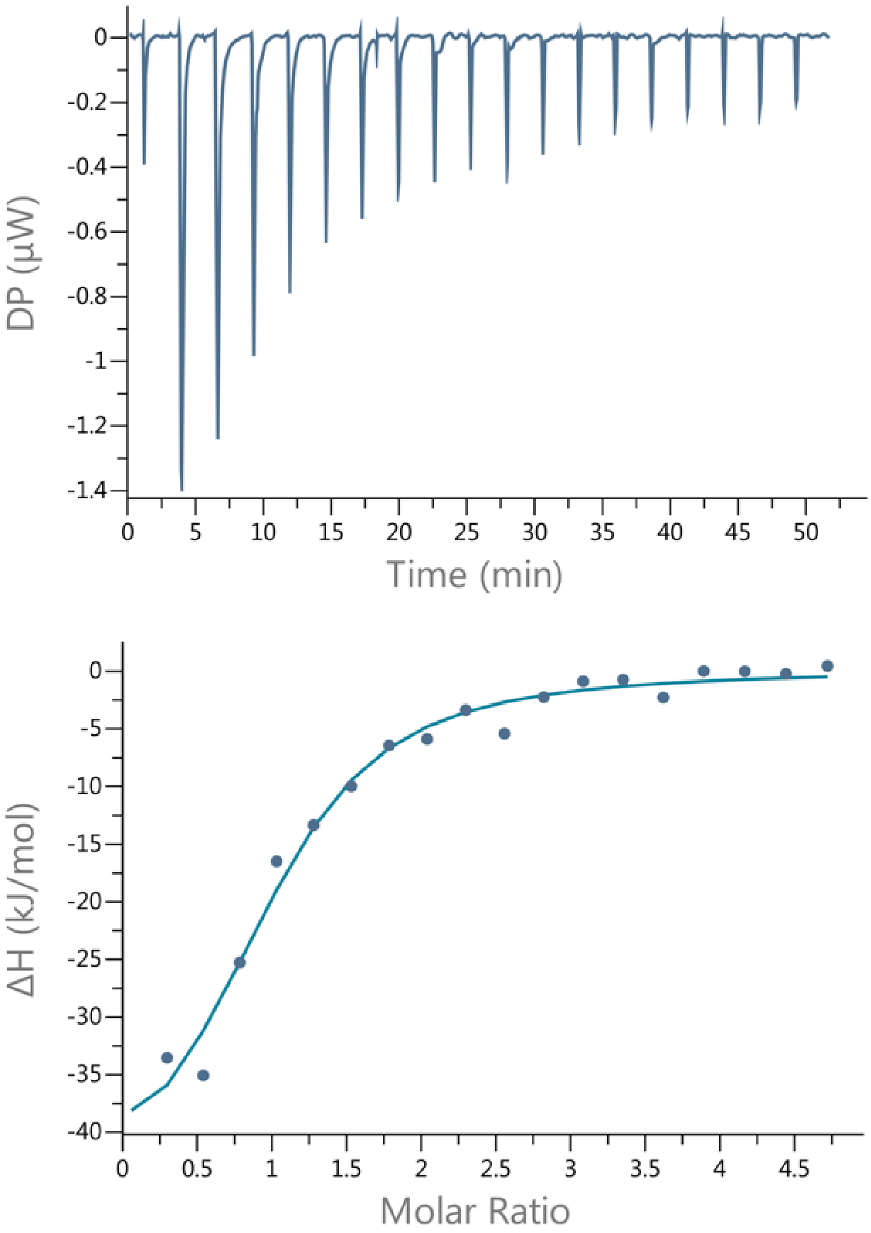

**Protein:** 10μM NMB 39		**Metal:** 250μM Co(II)
**N:** 0.949± 0.07		**K_D:** 2.17 ± 0.89 μM
**ΔH:** -47.4 ± 7.0 kJ/mol	**-TΔS:** 15.1 kJ/mol	**ΔG:** -31.5 kJ/mol

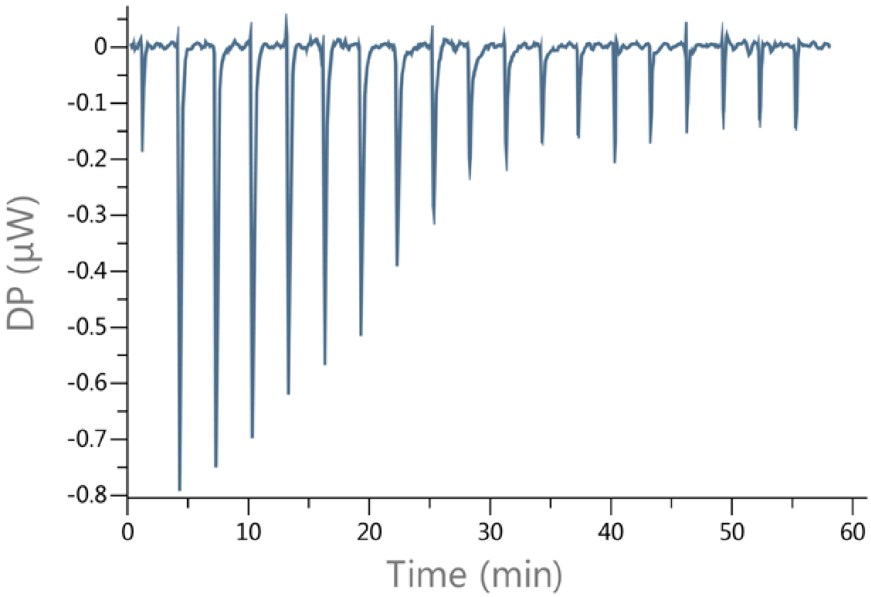

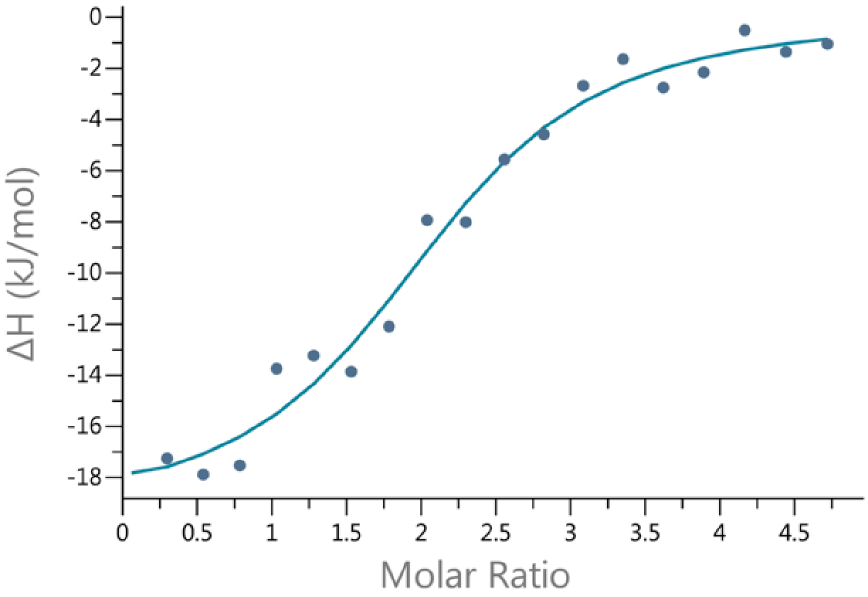

**Protein:** 10μM NMB 39		**Metal:** 250μM Cu(II)
**N:** 2.05 ± 0.10		**K_D:** 1.92 ± 0.82 μM
**ΔH:** -19.6 ± 2.1 kJ/mol	**-TΔS:** -13.1 kJ/mol	**ΔG:** -32.7 kJ/mol

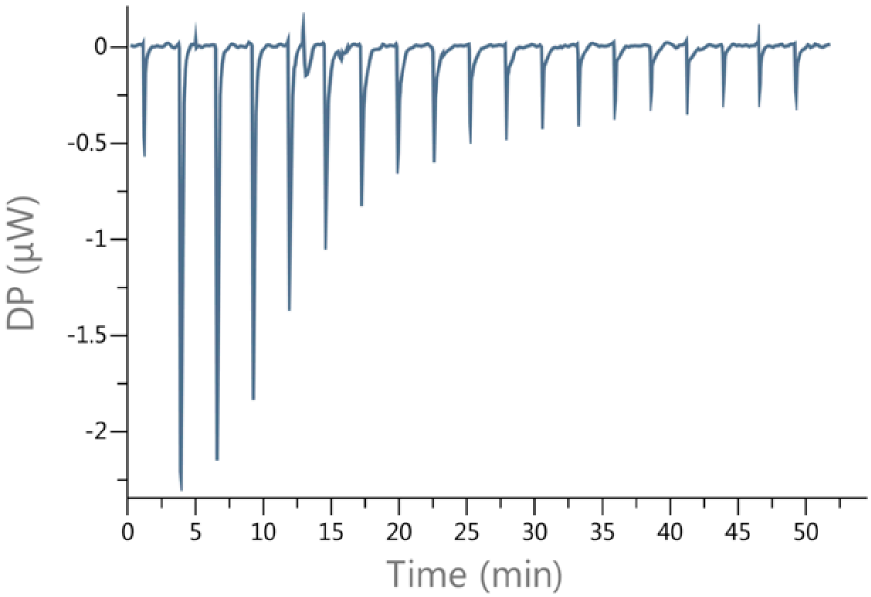

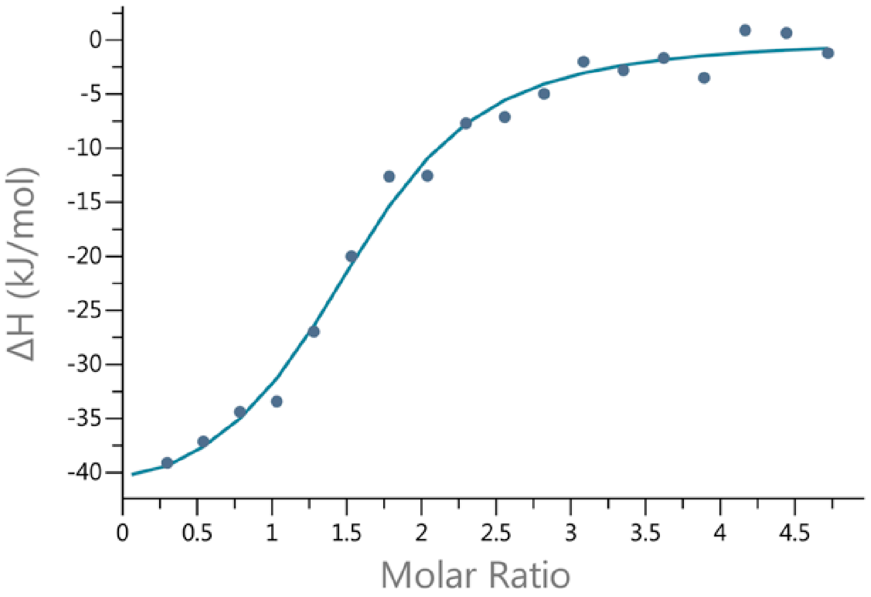

**Protein:** 10μM NMB 39		**Metal:** 250μM Zn(II)
**N:** 1.51 ± 0.05		**K_D:** 1.50 ± 0.42 μM
**ΔH:** -44.5 ± 3.0 kJ/mol	**-TΔS:** 11.2 kJ/mol	**ΔG:** -33.3 kJ/mol

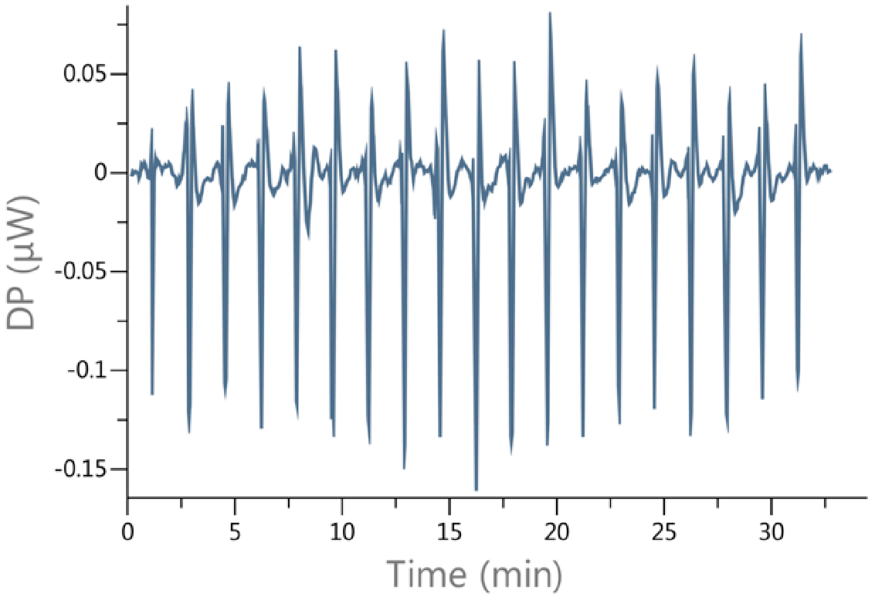

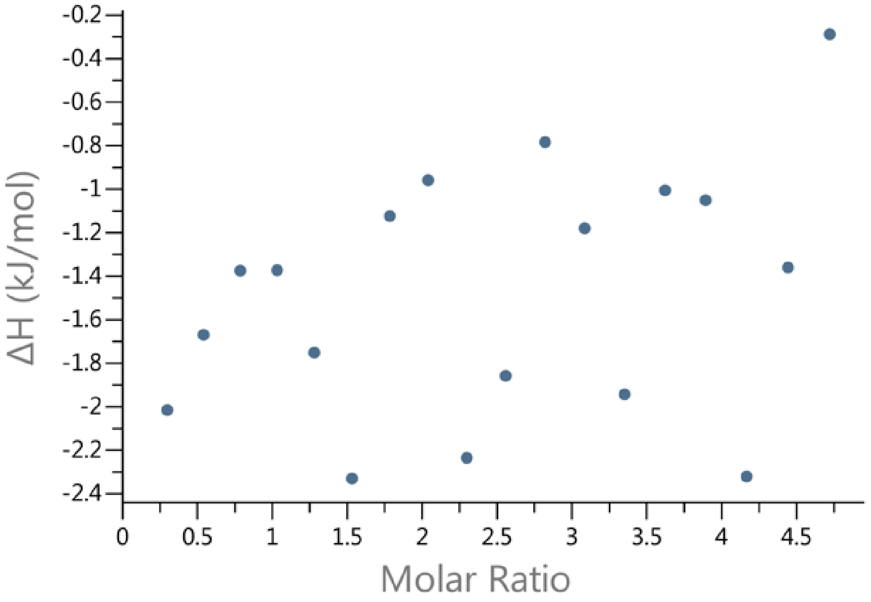

**Protein:** 10µM HisZero	**Metal:** 250µM Co(II)
**Raw Heat Average: -**1.486 ± 0.573 kJ/mol	

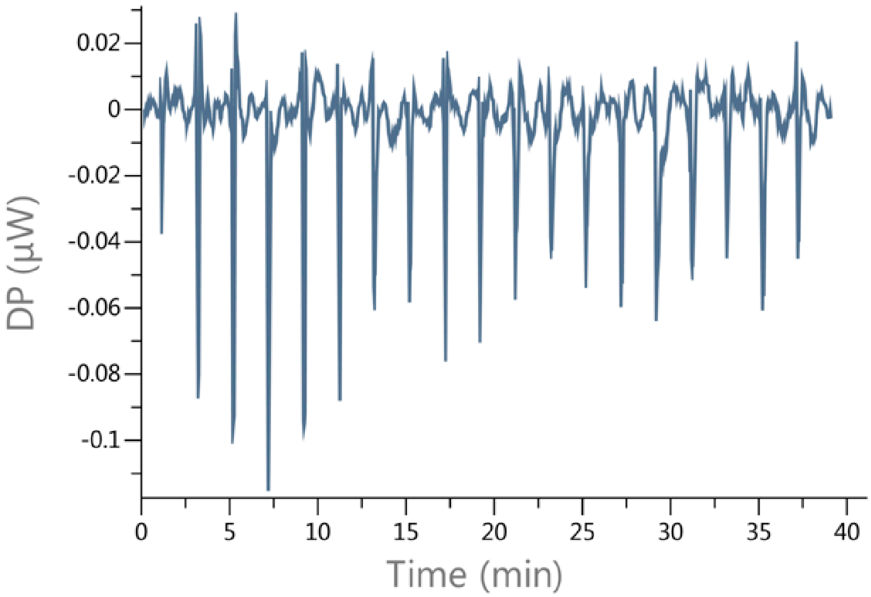

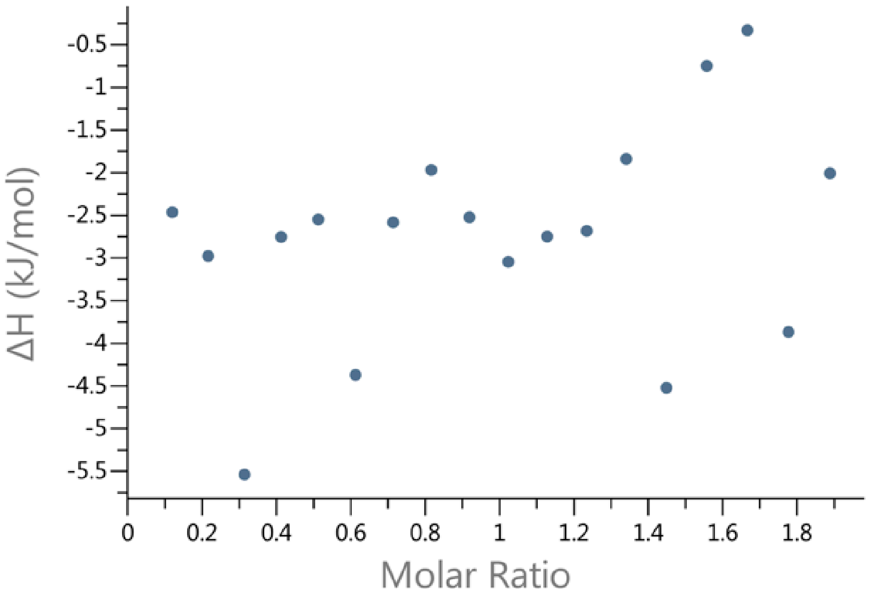

**Protein:** 10µM HisZero	**Metal:** 100µM Cu(II)
**Raw Heat Average:** -2.7706 ± 1.2599 kJ/mol	

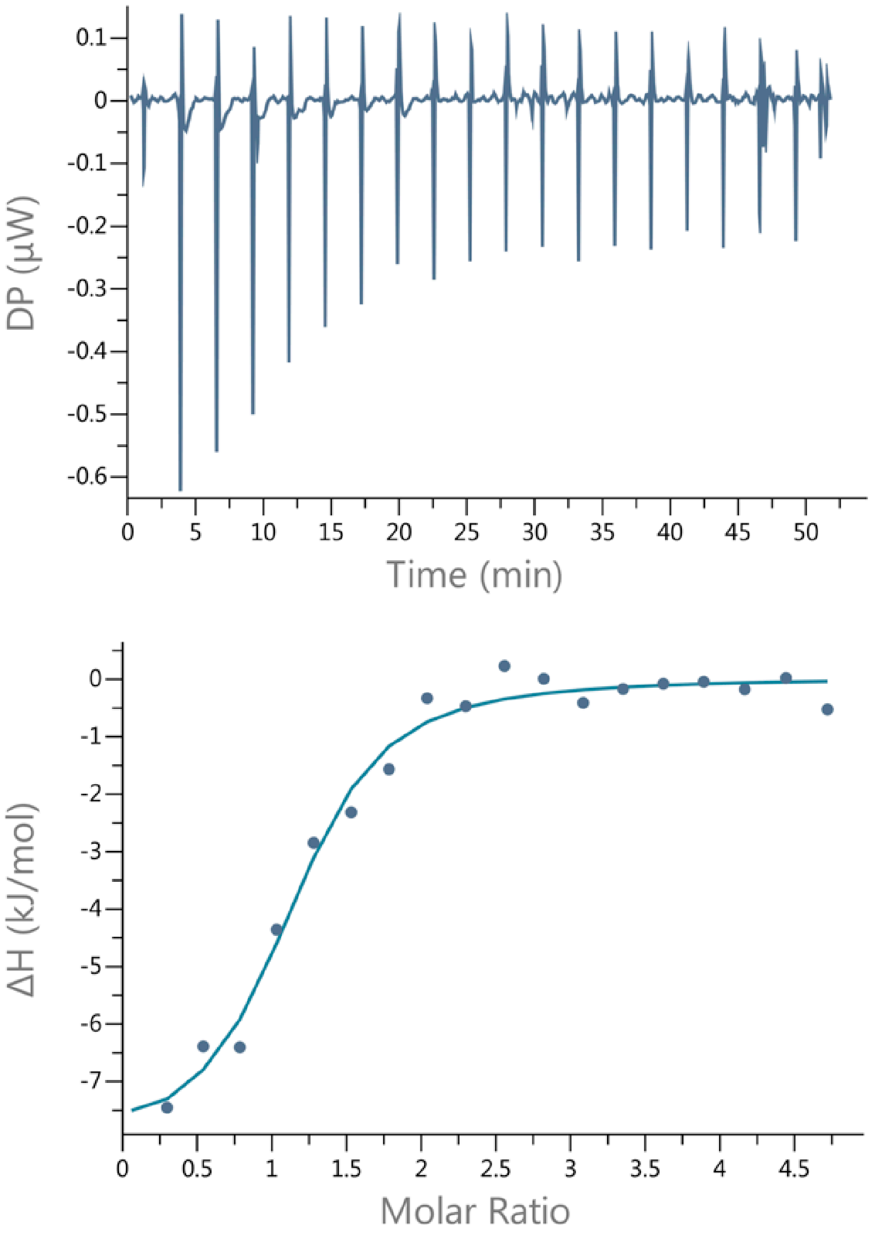

**Protein:** 10μM HisZero		**Metal:** 250μM Zn(II)
**N:** 1.09 ± 0.05		**K_D:** 0.961± 0.350 μM
**ΔH:** -8.23 ± 0.665 kJ/mol	**-TΔS:** -26.1 kJ/mol	**ΔG:** -34.4 kJ/mol

Amino Acid Abundance-Affinity Correlation:

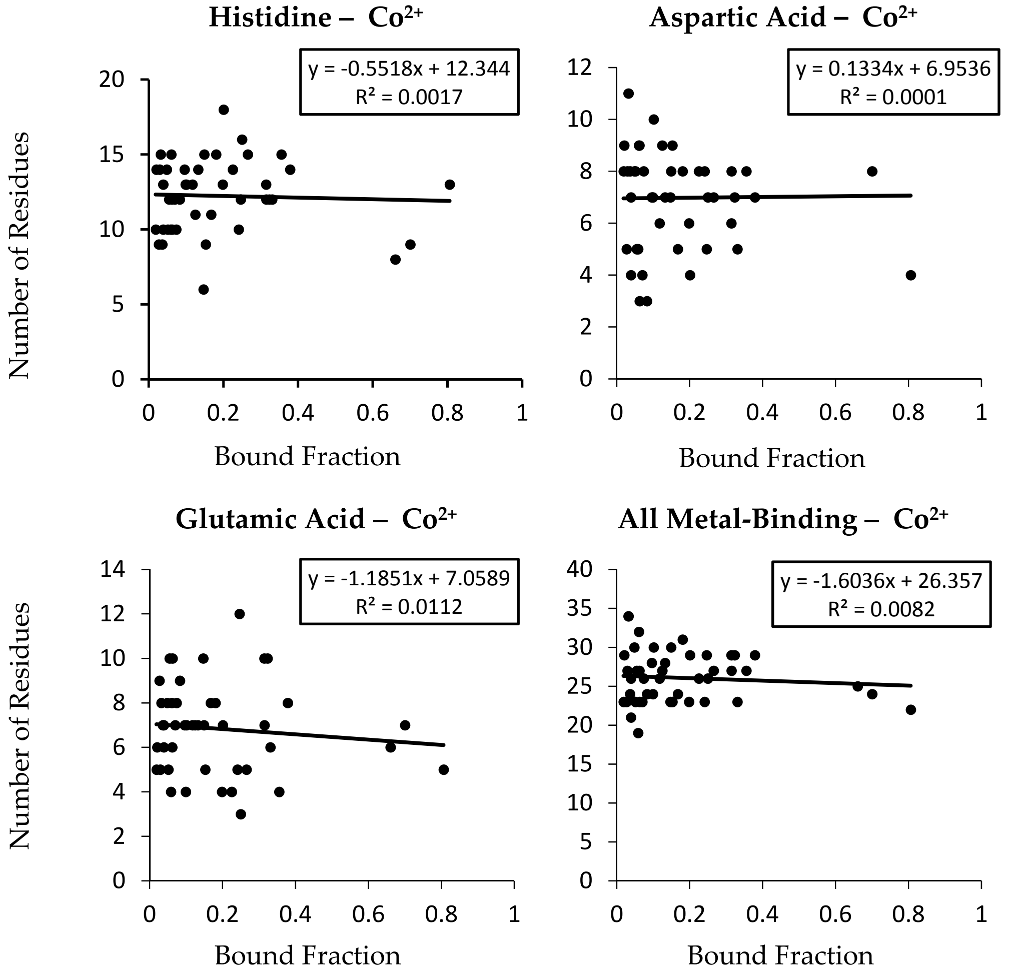

Figure S3: Lack of correlation between the abundance of metal binding residues (His, Asp, or Glu) and the observed binding to cobalt immobilized on a bead. The percentage of metal bound (i.e. remaining on the bead after stringent washes) is on the x axis, and the number of potential metal-binding residues is on the y axis.

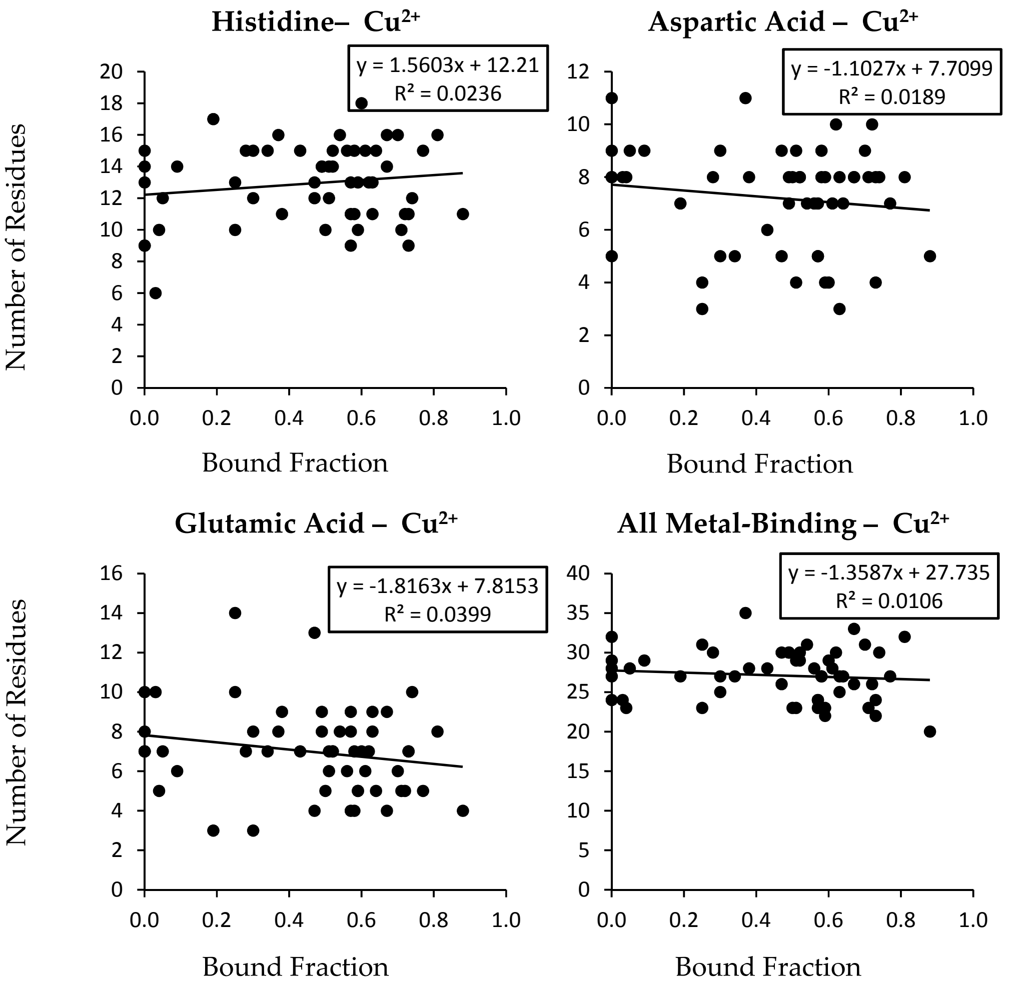

Figure S4: Lack of correlation between the abundance of metal binding residues (His, Asp, or Glu) and the observed binding to copper immobilized on a bead. The percentage of metal bound (i.e. remaining on the bead after stringent washes) is on the x axis, and the number of potential metal-binding residues is on the y axis.

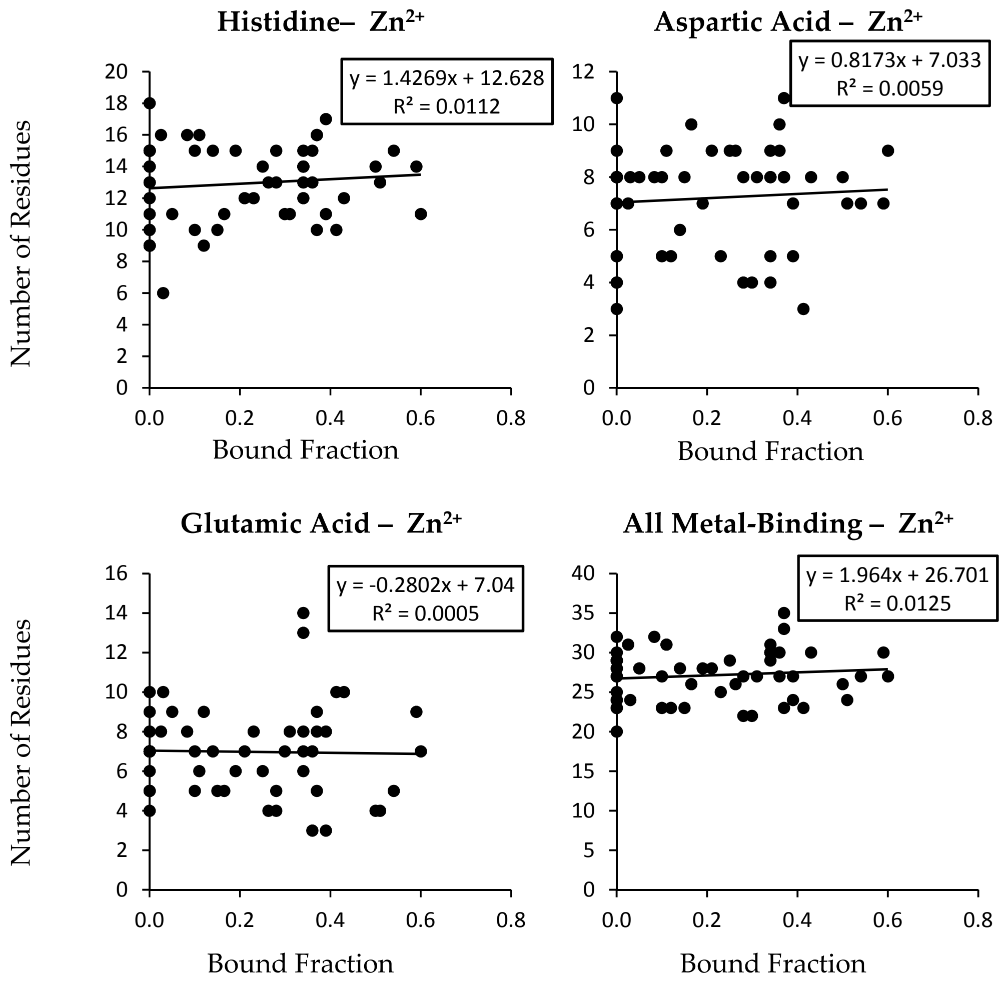

Figure S5: Lack of correlation between the abundance of metal binding residues (His, Asp, or Glu) and the observed binding to zinc immobilized on a bead. The percentage of metal bound (i.e. remaining on the bead after stringent washes) is on the x axis, and the number of potential metal-binding residues is on the y axis.
